# ALDH1A3 promotes invasion and metastasis in triple‐negative breast cancer by regulating the plasminogen activation pathway

**DOI:** 10.1002/1878-0261.13528

**Published:** 2023-10-09

**Authors:** Alamelu G. Bharadwaj, Meghan E. McLean, Margaret L. Dahn, Hannah F. Cahill, Marie‐Claire D. Wasson, Raj Pranap Arun, Olivia L. Walker, Brianne M. Cruickshank, Wasundara Fernando, Jaganathan Venkatesh, Penelope J. Barnes, Gillian Bethune, Gregory Knapp, Lucy K. Helyer, Carman A. Giacomantonio, David M. Waisman, Paola Marcato

**Affiliations:** ^1^ Department of Pathology Dalhousie University Halifax Canada; ^2^ Department of Surgery Dalhousie University Halifax Canada; ^3^ Department of Biochemistry and Molecular Biology Dalhousie University Halifax Canada; ^4^ Department of Microbiology and Immunology Dalhousie University Halifax Canada; ^5^ Nova Scotia Health Authority Halifax Canada

**Keywords:** ALDH1A3, invasion, metastasis, plasmin, tPA, triple‐negative breast cancer

## Abstract

Aldehyde dehydrogenase 1A3 (ALDH1A3) is a cancer stem cell marker that promotes metastasis. Triple‐negative breast cancer (TNBC) progression has been linked to ALDH1A3‐induced gene expression changes. To investigate the mechanism of ALDH1A3‐mediated breast cancer metastasis, we assessed the effect of ALDH1A3 on the expression of proteases and the regulators of proteases that degrade the extracellular matrix, a process that is essential for invasion and metastasis. This revealed that ALDH1A3 regulates the plasminogen activation pathway; it increased the levels and activity of tissue plasminogen activator (tPA) and urokinase plasminogen activator (uPA). This resulted in a corresponding increase in the activity of serine protease plasmin, the enzymatic product of tPA and uPA. The ALDH1A3 product all‐trans‐retinoic acid similarly increased tPA and plasmin activity. The increased invasion of TNBC cells by ALDH1A3 was plasminogen‐dependent. In patient tumours, ALDH1A3 and tPA are co‐expressed and their combined expression correlated with the TNBC subtype, high tumour grade and recurrent metastatic disease. Knockdown of tPA in TNBC cells inhibited plasmin generation and lymph node metastasis. These results identify the ALDH1A3–tPA–plasmin axis as a key contributor to breast cancer progression.

## Graphical abstract

Aldehyde dehydrogenase 1A3 (ALDH1A3) regulates expression of tissue plasminogen activator (PLAT/tPA), leading to increased invasion and metastasis of breast cancer cells. ALDH1A3 generates all‐trans retinoic acid (ATRA) from retinal. ATRA induces expression of protease PLAT/tPA. Secreted tPA activates plasminogen. Plasmin degrades the extracellular matrix, facilitating breast cancer cell invasion and metastasis, including metastasis to lymph nodes. Created with BioRender.com.

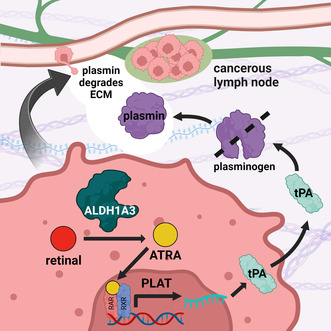

**Abbreviations**
AA: antibiotic antimycotic
ADAM: a disintegrin and metalloprotease
ADAMTS: a disintegrin and metalloprotease with thrombospondin motif
ALDH: aldehyde dehydrogenase
ALDH1A3: aldehyde dehydrogenase 1A3
ATCC: American Type Culture Collection
ATRA: all‐trans retinoic acid
CM: conditioned media
CpG: 5′—C—phosphate—G—3′
DMEM: Dulbecco's Modified Eagle Medium
GAPDH: glyceraldehyde 3‐phosphate dehydrogenase
GDAC: Genome Data Analysis Centers
GEO: Gene Expression Omnibus
H&E: haematoxylin and eosin
HER2: human epidermal growth factor 2
IgG‐HRP: immunoglobulin G conjugated to horse radish peroxidase
METABRIC: Molecular Taxonomy of Breast Cancer International Consortium
MMP: matrix metalloproteinase
NOD/SCID: nonobese diabetic/severe combined immunodeficiency
PAI‐1: plasminogen‐activator‐inhibitor 1
PAI‐2: plasminogen‐activator‐inhibitor 2
PLAT: plasminogen activator, tissue type
PLAU: plasminogen activator, urokinase
pNA: *p*‐nitroanilide
QEII HSC: Queen Elizabeth II Health Science Center
RAR: retinoic acid receptor
RARE: retinoic acid receptor element
REB: Research Ethics Board
RNA‐seq: RNA sequencing
RSEM: RNA‐seq by expectation–maximization
RT‐qPCR: reverse‐transcription quantitative PCR
RXR: retinoid X receptor
SERPINB2: serpin family B member 2
shRNA: short hairpin RNA
siRNA: small interfering RNA
STR: short tandem repeat
TCGA: The Cancer Genome Atlas
TIMP3: tissue inhibitor of metalloproteinase 3
TNBC: triple‐negative breast cancer
tPA: tissue plasminogen activator
TSS: transcription start site
uPA: urokinase plasminogen activator

## 1 Introduction

Breast cancer is the most common cancer in women and mortality typically results from metastatic disease that does not respond to therapy [1]. This emphasizes the need for an enhanced and comprehensive understanding of the molecular factors involved in breast cancer metastasis to inform new treatment strategies. These factors include aldehyde dehydrogenase 1A3 (ALDH1A3), a cancer‐promoting enzyme associated with cancer stem cells, poor prognosis, and the more aggressive triple‐negative breast cancer (TNBC) subtype [2, 3, 4, 5]. Functionally, ALDH1A3 promotes tumour growth, invasion and metastasis, and contributes to chemoresistance [3, 4, 5, 6, 7, 8, 9, 10, 11, 12, 13, 14]. ALDH1A3 is a member of the ALDH superfamily genes, which in general oxidize aldehydes arising from lipid peroxidation, amino acid catabolism and xenobiotics [15]. Furthermore, ALDH1A3 generates the cell signalling molecule all‐trans retinoic acid (ATRA) from vitamin A metabolite retinal [16]. ATRA binds to multiple nuclear receptors leading to expression changes in hundreds of genes, resulting in differentiation, cell cycle arrest, or cell proliferation. ALDH1A3's cancer‐promoting activities in TNBC are least in part related to its generation of ATRA and subsequent gene expression changes [3], although how these gene expression changes affect its invasion and metastasis‐promoting function is unclear.

Here in we investigate the molecular basis of ALDH1A3‐mediated invasion and metastasis in TNBC. The metastatic cascade begins with tumour cells invading the surrounding extracellular matrix. Cancer cell surface proteolysis is essential for invasion and is an early step in metastasis. Given the critical role of proteases in the degradation of the extracellular matrix, we investigated the possibility that ALDH1A3 might play a role in regulating proteases that play a role in breast cancer progression. These include extracellular proteases, such as serine (plasmin), cysteine, aspartic and matrix metalloproteases (MMPs) that are known to degrade and remodel the extracellular matrix [17]. We found evidence that ALDH1A3 regulates key players in the plasminogen activation pathway.

Plasmin cleaves fibrin in the extracellular matrix and activates pro‐MMPs [18, 19, 20], accelerating extracellular matrix remodelling required for invasion [21, 22, 23]. Plasminogen is a zymogen synthesized and secreted by the liver [24]. The activation of plasminogen to the serine protease plasmin is mediated by activators such as tissue plasminogen activator (tPA), and the urokinase plasminogen activator (uPA), and its receptor. The activation is tightly regulated by the expression of plasminogen‐activator‐inhibitors (PAI‐1 and PAI‐2) that inhibit the tPA and uPA activity. In addition, cell surface plasminogen receptors accelerate the conversion of plasminogen to plasmin by tethering the plasminogen to the cell surface and co‐localizing it with its activators [25, 26, 27]. The increased ability of the cancer cell to generate plasmin is directly correlated with increased invasive and metastatic potential [28, 29]. Plasmin functions in invasion and metastasis by directly degrading the extracellular matrix proteins such as laminin and fibronectin and indirectly by activating MMPs [30].

Our data suggest that ALDH1A3 transcriptionally regulates tPA, uPA and PAI‐2, leading to ALDH1A3‐dependent plasmin activity in TNBC. ALDH1A3‐mediated invasion was impeded in the absence of plasminogen. Mechanistically, we connected increased tPA and plasmin activity in TNBC cells to ATRA, a transcriptional regulator which is produced by ALDH1A3. In breast cancer patient tumour tissues, ALDH1A3 and tPA were significantly co‐expressed and associated with features of aggressive disease. Finally, we show that tPA contributes to metastasis development in an orthotopic xenograft tumour model. Together, this study demonstrates that the ALDH1A3‐tPA‐plasmin axis is a novel cell signalling pathway that promotes progressive breast cancer disease.

## 2 Materials and methods

### 2.1 Cell culture and reagents

Cancer cell lines were obtained from the American Type Culture Collection (ATCC, Manassas, VA, USA). MDA‐MB‐231 (RRID: CVCL_0062), MDA‐MB‐468 (RRID: CVCL_0419), and HEK293T (RRID: CVCL_0063) cells were grown in Dulbecco's Modified Eagle Medium (DMEM; ThermoFisher) supplemented with 10% FBS (ThermoFisher, Mississauga, Canada) and antibiotic antimycotic (AA; ThermoFisher). MDA‐MB‐436 (RRID: CVCL_0623) cells were grown in Leibovitz's Medium (L‐15; ThermoFisher) supplemented with 10% FBS, AA, 10 μg·mL^−1^ human insulin (Millipore Sigma, Oakville, Canada), and 16 μg·mL^−1^ l‐glutathione (ThermoFisher). Cells were cultured in a humidified 37 °C incubator with 5% CO_2_, except for MDA‐MB‐436 cells which was cultured without the addition of CO_2_. The cell lines have been authenticated in the past 3 years by isolation of genomic DNA and performance of short tandem repeat profiling (STR) technology by Applied Biological Materials Inc. (abm, Richmond, Canada). Abm follows the International Cell Line Authentication Committee (ICLAC) standard ASN‐002 in preforming the STR analysis. We regularly perform assessment for mycoplasma contamination using the MycoAlert® Mycoplasma Detection Kit (Lonza, Kingston, Canada) and confirm that all experiments were conducted with mycoplasma‐free cells.

Cell experiments including ATRA treatments (100 nm; Millipore Sigma) were conducted for 24 h. For experiments with serum‐free conditioned media (CM), MDA‐MB‐231 cells were cultured using DMEM, without phenol red (21‐063‐029; ThermoFisher), and sodium pyruvate (ThermoFisher) while MDA‐MB‐436 cells were cultured in Leibovitz's L‐15 Medium, with no phenol red (21083027; ThermoFisher). For assays with plasminogen depleted media, plasminogen was depleted from FBS by passing the FBS through a lysine sepharose column, which allows plasminogen to bind to the column. The flow‐through from the lysine sepharose column was collected and filter sterilized using 0.2 μm filters (ThermoFisher).

ALDH1A3‐over expression for MDA‐MB‐231 and was generated as described previously [2, 3] and validated by western blotting again here. We also generated short hairpin RNA (shRNA) knockdowns in MDA‐MB‐436 and MDA MB‐468 cells using the retroviral vector pSMP (Open Biosystems, Huntsville, AL, USA) with either the shRNAmir scramble sequence or shRNAmir sequences specific to ALDH1A3 (Table S1) following standard procedures. The retroviral supernatants were applied to cultured MDA‐MB‐436 and MDA MB 468 cells. Where relevant, lentiviral shRNA knockdown clones of PLAT were generated using the pGipZ vector (Dharmacon, Lafayette, Colorado, USA) packaged in HEK293T cells following standard protocols and listed in Table S1. Clones were selected by adding 1.5 μg·mL^−1^ puromycin and subsequently maintained in 0.25 μg·mL^−1^ puromycin media. Transient knockdown of PLAT was achieved by applying siRNA sequences (Integrated DNA Technologies, Coralville, IA, USA) with lipofectamine 2000 (ThermoFisher) to cells as per manufacturer's protocol. The siRNA sequences are listed in Table S1.

### 2.2 Reverse‐transcriptase quantitative PCR

For gene expression analysis by reverse‐transcriptase quantitative PCR (RT‐qPCR) cells were collected in Trizol (ThermoFisher) and RNA was purified using a PureLink RNA kit (ThermoFisher) following the manufacturer's instructions. Equal amounts of purified RNA were then reverse‐transcribed to cDNA using iScript (Bio‐Rad, Mississauga, Canada) as per the manufacturer's instructions. Diluted cDNA was used in RT‐qPCR reactions with gene‐specific primers (Table S2) and SsoAdvanced Universal SYBR Supermix (Bio‐Rad) as per manufacturer's instructions with a CFX96 or CFX384 Touch Real‐Time PCR Detection System (Bio‐Rad). Standard curves were generated for each primer set and primer efficiencies were incorporated into the cfx manager software (Bio‐Rad). Relative expression of genes in cells was quantified using the ΔΔ*C*_t_ method of the cfx manager Software (Bio‐Rad), where gene‐of‐interest quantification was normalized to at least two reference genes (Table S2) and then made relative control cells mRNA levels.

### 2.3 Western blotting

Equal concentration protein from cell lysates and CM were separated by 12% SDS/PAGE gels. The proteins were transferred to nitrocellulose or PVDF membranes and blocked with 5% skim milk. The membranes were probed using anti‐human ALDH1A3 (OTI4E8; OriGene Technologies, Rockville, MD, USA), tPA (ab227069; Abcam, Toronto, Canada), uPA (ab24121; Abcam), and PAI‐2 (ab47742; Abcam) antibodies. Anti‐rabbit IgG‐HRP (7074S; Cell Signaling Technology) secondary antibody was used for tPA, uPA, and PAI‐2 while anti‐mouse IgG‐HRP (7076S; Cell Signaling Technology, Danvers, MA, USA) secondary antibody was used for ALDH1A3. Immuno‐reactive proteins were detected by chemiluminescence (using Clarity ECL blotting substrate (Bio‐Rad)) and visualized with images captured with a ChemiDoc Imager (Bio‐Rad). Total protein was used as a loading control.

### 2.4 Immunofluorescence analysis

Patient tumour core biopsy tissues taken post‐surgery from patients who were diagnosed with breast cancer at the Queen Elizabeth II Health Sciences Centre (QEII HSC) in Halifax, NS, Canada, were analysed for immunofluorescence staining. All study methodologies with patient tissues conformed to the standards set by the Declaration of Helsinki. The study methodologies were approved by the IWK‐Research Ethics Board and the Nova Scotia Heath Research Ethics Board. The research ethics board (REB) numbers for the approved protocols are 1007109 (IWK) and 1028015 (Nova Scotia Health). The experiments contain patient tumours samples from 2011 to 2019, with follow up clinical data until 2022. In all experiments, patient samples were obtained from individuals who had a clear understanding of the study and provided written consent, except for patient samples collected between 2011 and 2014 under the approval of REB #1028015, for which a waiver of consent was granted. The waiver was granted for two reasons. Since they were treated and diagnosed in 2014 or earlier, we lack relationships with patients and obtaining consent retroactively provided insurmountable difficulties for our group. Second, only a small sample of archived FFPE material post‐diagnosis was used in the study and there was no impact on the patient with respect to treatment choices or prognosis.

Tissues were formalin‐fixed and paraffin‐embedded. Staff pathologists at the QEII HSC conducted a standard pathological assessment of patient tumours (Table S3). Five micrometre sections cut from paraffin‐embedded blocks were deparaffinized for staining. Post antigen retrieval, and blocking, slides were stained with the above‐described antibodies. Secondary IgG antibodies specific to the species for dual labelling were conjugated to either goat anti‐rabbit Alexa 488 (A32731; ThermoFisher) or goat anti‐mouse Alexa 555 (A32727; ThermoFisher). Nuclei were stained with DAPI (D1306; ThermoFisher). Stained slides were mounted (P36982; ThermoFisher) and images were captured with a Zeiss LSM 880 with AiryScan laser scanning confocal microscope (Carl Zeiss Canada Ltd., Toronto, Canada).

To quantify the number of positive tumour cells in each sample, multiple sections were stained to ensure that representative areas of the entire tissue were assessed. Between three to five random images were captured of each section. To estimate the percentages of positive tumour cells, a colocalization image creator and colocalization object counter imagej (developed by the National Institutes of Health (Bethesda, MD, USA) and the Laboratory for Optical and Computational Instrumentation, University of Wisconsin, Madison, WI, USA) plugins were used to semi‐automatically count positively stained cells. This information was then used to calculate the average percentage of ALDH1A3+, tPA+, uPA+, PAI‐2+, ALDH1A3+/tPA+, ALDH1A3+/uPA+, and ALDH1A3+/PAI‐2− cells within tumour samples.

### 2.5 Cell surface plasminogen activation assay

Cells were seeded at a density of 30 000 cells/well in 96‐well plates (Corning, Corning, NY, USA) overnight and washed three times with incubation buffer (Hanks balanced salt solution containing 3 mm CaCl_2_ and 1 mm MgCl_2_; ThermoFisher). For experiments with siRNA, the cells were transfected with the siRNA 48 h before commencement of the experiment. Cells were then incubated with 0.5 μm Glu‐plasminogen (Molecular Innovations, Novi, MI, USA), for 15–20 min at 37 °C before the addition of 500 μm plasmin chromogenic substrate (S2251; Chromogenix, Diapharma Group, West Chester, OH, USA). Plasmin activity was measured spectrophotometrically (405 nm) taking readings every 2 min for 2 h. Time course data were analysed according to the equation describing the rate of *p*‐nitroanilide (*p*‐NA) production *A*405 nm = *B* + *Kt*2, where *K* is the rate constant for the acceleration of *p*‐NA generation and *B* is the *y*‐intercept. Under our experimental conditions, *K* is proportional to the initial rate of plasmin formation from plasminogen.

### 2.6 tPA and uPA activation assays

Cells were seeded at 2–3 × 10^6^ cells per plate and serum starved after 24 h of incubation. For experiments with siRNA, the cells were transfected with the siRNA for 72 h before measuring tPA activity in CM. Specifically for tPA activity in CM, siRNA transfected cells were conditioned in serum‐free media after 24 h post transfection for 48 h, before determination of the activities. The CM was harvested after 48–72 h of conditioning and then centrifuged at 2000 ***g*** for 15–20 min to remove cell debris. The CM was concentrated using Amicon centrifugation filter devices (ThermoFisher) with a 30 kD molecular weight cut‐off. The protein concentration in the CM was determined using a BCA assay and equal protein amounts (50 μg) were used for tPA and uPA activity assays. The tPA activity was determined using 0.5 mm tPA substrate S2288 (Diapharma Group), and uPA activity was determined using 0.5 mm of uPA substrate cs‐uk‐dpg444‐25 (Diapharma). Briefly, the CM and respective substrates were mixed and activity was measured spectrophotometrically (405 nm) taking readings every 2 min for 2 h at 37 °C. Time course data were analysed according to the equation describing the rate of *p*‐NA production *A*405 nm = *B* + *Ktt*, where *K* is the rate constant for the acceleration of *p*‐NA generation and *B* is the *y*‐intercept.

### 2.7 Transwell invasion assay

2.5 × 10^4^ cells were seeded in the upper well of either a Matrigel‐coated invasion chamber (Corning) or an uncoated migration chamber (Corning) with 8 μm pore size in serum‐free media, with 10% FBS containing media in the bottom well as a chemoattractant. For experiments with siRNA, the cells were transfected with the siRNA for 24 h before commencement of the experiment. For the experiments with plasminogen‐stripped FBS, the bottom chamber contained 10% plasminogen‐stripped FBS as a chemoattractant ±0.25 μm plasminogen (Molecular Innovations). We added plasminogen to bottom chamber along with plasminogen‐stripped FBS to replicate the “unstripped” regular FBS, which is normally used in transwell invasion assays and has plasminogen among its components. The FBS in the bottom well acts as a chemoattractant, but it also freely diffuses into the top well. Like other FBS components, plasminogen in the bottom well would diffuse into the upper chamber and be accessible to cells to activate into plasmin. After 24 h, migrated or invaded cells that had crossed the chamber membrane were fixed in methanol and stained with 0.05% crystal violet. Transversed cells were counted in 4–5 fields of view per chamber at 20× using a Motic AE31E light microscope (Motic Microscopes, Vancouver, Canada). The percent invasion was determined via the following equation:

$$\;\%\;\text{Invasion}=\frac{\text{mean number of cells invaded through Matrigel}-\text{coated transwell}}{\text{mean number of cells migrated through uncoated transwell}}\times 100.$$

### 2.8 Orthotopic tumour xenograft experiment

All experiments followed guidelines set by the Canadian Council on Animal Care and were performed according to a study and protocol approved by Dalhousie University Committee on Laboratory Animals (protocol 21‐011). Nonobese diabetic/severe combined immunodeficiency (NOD/SCID) female mice from Charles River Laboratories were housed in ventilated racks, in sterilized barrier cages (5 mice per cage), and were fed sterilized food and water *ad‐libitum*, with 12 h light and dark cycles. The 7‐week‐old NOD/SCID female mice were orthotopically injected in the mammary fat pad four with 2 × 10^6^ cells of MDA‐MB‐231, with or without PLAT (tPA) knockdown along with a 1 : 1 ratio of high concentration Matrigel (Corning). Primary tumour growth was quantified (length × length × height/2). At termination, the lungs, the axillary and inguinal lymph nodes, and tumours were harvested for analysis. The lungs (minus the left lung lobe that was stored in RNAlater reagent for later analysis by RT‐qPCR) and nodes were fixed, paraffin‐embedded and sectioned (5 μm) for metastasis visualization by haematoxylin and eosin (H&E) or stained by immunofluorescence with a pan‐cytokeratin antibody (M3515; Agilent Technologies Inc., Dako, Santa Clara, CA, USA) and secondary anti‐mouse antibody described above. H&E‐stained sections were imaged using an Aperio Scanning system (Leica Biosystems, Concord, ON, Canada) at 2× magnification and a further 10× zoom magnification of cropped images was done as indicated in legends as indicated.

### 2.9 Quantification disseminated MDA‐MB‐231 cells in the lungs of mice by human‐specific GAPDH RT‐QPCR

To quantify the number of MDA‐MB‐231 cells in the left lung lobe from the above‐described experiment, we used our previously published method that can accurately quantify between 100 and 1 000 000 disseminated human cells in mouse lungs [31], Briefly, the RNA was extracted from the lung lobes and RT‐qPCR performed as described above. In the RT‐qPCR assay, we used our previously validated human‐specific and mouse GAPDH primers [31] (Table S2). The number of MDA‐MB‐231 cells detected in the lung lobes was calculated based on a standard curve generated from RNA extracted from naïve lung lobes that had been spiked with increasing numbers of MDA‐MB‐231 cells (ranging from 10 to 1 000 000 cells).

### 2.10 Transcriptome, 450K methylation, and patient dataset analysis

Microarray gene expression data for MDA‐MB‐231 control and overexpressing ALDH1A3 (*n* = 3; GSE103426) was analysed. Breast cancer (Molecular Taxonomy of Breast Cancer International Consortium (METABRIC), TNBC, hormone receptor positive, or HER2+ patient tumours, or normal adjacent tissues within the dataset) and Breast Invasive Carcinoma (The Cancer Genome Atlas, TCGA, Cell 2015; TNBC, hormone receptor positive, or HER2+ patient tumours within the dataset) clinical data and RNA sequencing (RNA‐seq) log_2_ V2 RSEM and gene array expression data were accessed via cBioportal [32, 33]. CpG methylation of PLAT and PLAU in MDA‐MB‐231 and MDA‐MB‐436 cell lines was determined by analysing 450K methylation data (GSE78875). The corresponding methylation of specific CpG sites in PLAT and PLAU was accessed from the 450K methylation data from TCGA Firehose cohort obtained from the Broad Institute Genome Data Analysis Centers (GDAC) portal.

### 2.11 Statistical analyses

All statistical analyses were calculated in graphpad prism 9. (GraphPad Software, Boston, MA, USA) *T* tests were performed when two experimental conditions were compared. In cases when multiple conditions were tested, one‐way ANOVA analyses were performed followed by multiple comparisons analysis. Pearson and Spearman correlation coefficient analyses were conducted on gene expression correlations of patient tumour data. A log‐rank test was conducted on Kaplan–Meier overall and progression‐free survival curve analyses. *P* values are represented as follows: *< 0.05, **< 0.01, ***< 0.001, ****< 0.0001. Statistical tests and significance are indicated in all figure captions.

## 3 Results

### 3.1 ALDH1A3 is co‐expressed with factors in the plasminogen activation pathway in TNBC

**Fig. 1:**
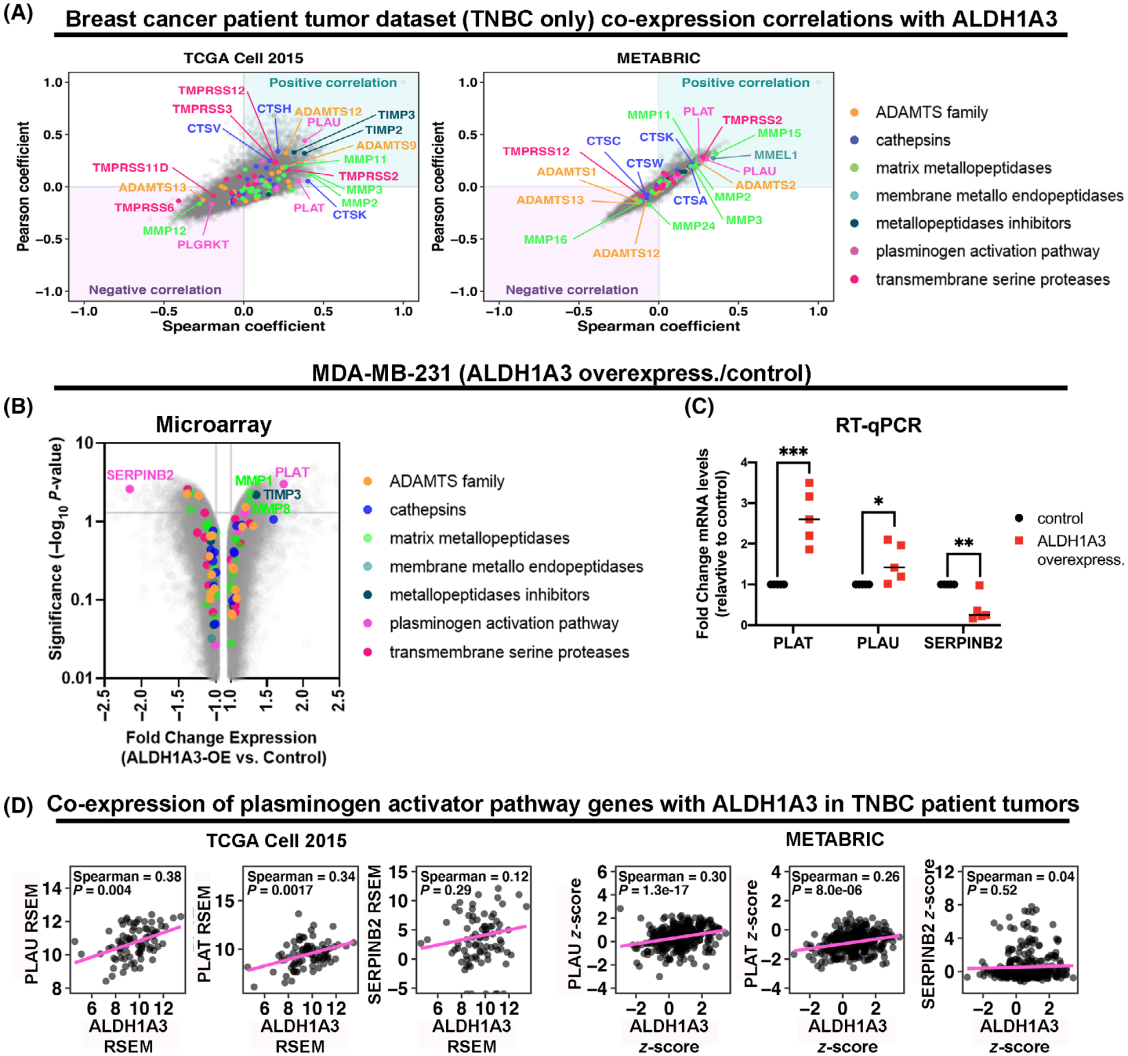
ALDH1A3 is co‐expressed with genes in the plasminogen activation pathway in TNBC. (A) Pearson and Spearmen coefficients were calculated based on the co‐expression of ALDH1A3 and all the genes in the subset of patients identified as TNBC in the METABRIC (*n* = 320); TCGA, Cell 2015 (*n* = 82); RNA‐Seq RSEM log_2_. Proteases or protease regulator genes are identified (Table S4). (B) Transcriptome analysis of MDA‐MB‐231 cells (ALDH1A3 overexpression versus control cells) completed by Affymetrix Human Gene 2.0ST Array (*n* = 3) identified differential expression of protease or protease regulator genes, the grey horizontal line indicates *P* < 0.05, by ANOVA method: ebayes. (C) Real time‐quantitative polymerase chain reaction (RT‐qPCR) of MDA‐MB‐231 cells (ALDH1A3 overexpression versus control cells), *n* = 5, significance determined by paired *t*‐test, and are indicated as follows: *< 0.05, **< 0.01, ***< 0.001. (D) Co‐expression analysis of ALDH1A3 versus PLAU (plasminogen activator, urokinase), PLAT (plasminogen activator, tissue type), and SERPINB2 (serpin family B member 2, also known as plasminogen‐activator‐inhibitor 2) in TNBC patient samples from TCGA Cell 2015 and METABRIC datasets. Spearman coefficient and adjusted *P* values are indicated. Significant *P* values are indicated as follows: *< 0.05, **< 0.01, ***< 0.001.

We have shown that ALDH1A3 mediates invasion and metastasis in TNBC, which is at least in part attributable to gene expression changes induced by ALDH1A3 [3]. Therefore, to investigate potential mechanisms for ALDH1A3‐mediated invasion, we assessed the TCGA RNAseq and METABRIC gene array data for gene co‐expression with ALDH1A3 in TNBC tumours. In the graphs, genes with high positive Spearman and Pearson correlations are positively co‐expressed with ALDH1A3, while genes with low negative Spearman and Pearson correlations are negatively co‐expressed with ALDH1A3 (Fig. 1A). We focused our analysis specifically on the 86 genes that are proteases or regulators of proteases known to mediate the degradation of the extracellular matrix in cancer progression (Fig. 1A, Table S4 lists the 86 genes). In both patient data sets, we noted significant positive co‐expression between ALDH1A3 and transmembrane serine protease 2 (TMPRSS2), MMP2, 3 and 11, and PLAT and PLAU that encode the plasminogen activators, tPA and uPA, respectively.

Although these co‐expression analyses in patient tumour data suggest a potential connection between ALDH1A3 and these proteases and protease regulators, it does not necessarily mean direct regulation of the genes by ALDH1A3. Therefore, we assessed the same 86 genes in our gene array data of TNBC MDA‐MB‐231 cells, with or without ALDH1A3 overexpression. Among the genes, the two most prominent ALDH1A3‐regulated genes in MDA‐MB‐231 cells are PLAT (encodes tPA), and SERPBINB2 (encodes PAI‐2, an inhibitor of tPA and uPA) (Fig. 1B). ALDH1A3 also upregulated MMP1 and MMP8; however, these were not highly co‐expressed with ALDH1A3 in the patient tumour data (Fig. 1A). We also noted that ALDH1A3 upregulated TIMP metallopeptidase inhibitor 3 (TIMP3) in MDA‐MB‐231 cells (Fig. 1B), and TIMP3 was highly co‐expressed with ALDH1A3 in the TCGA Cell 2015 patient tumour dataset (Fig. 1A). TIMP3 is a well‐known inhibitor of the MMPs and a disintegrin and metalloproteinases (ADAMs), and ADAM with thrombospondin motifs (ADAMTSs) proteins [34, 35]. The upregulation of TIMP3 by ALDH1A3 suggests the metalloproteases could be inhibited in high‐ALDH1A3 expressing cells making metalloprotease‐mediated invasion by ALDH1A3 in the cells less likely. Together, the patient tumour and cell line expression data most consistently implicated the plasminogen activation pathway could be important in ALDH1A3‐mediated invasion. We, therefore, confirmed the ALDH1A3‐dependent regulation of PLAT, PLAU, and SERPINB2 by RT‐qPCR (Fig. 1C) and visualized the co‐expression correlation of the genes with ALDH1A3 in the patient tumour data (Fig. 1D). Consistently, we noted generally similar co‐expression of these genes with ALDH1A3 in hormone receptor+ breast cancers (Fig. S1A), normal adjacent tissues (Fig. S1B) and TNBC cell lines (Fig. S1C). Interestingly, in human epidermal growth factor 2 overexpressing (HER2+) breast cancers, ALDH1A3 expression did not correlate with PLAT expression (Fig. S1D). The co‐expression of PLAT and PLAU with ALDH1A3 in TNBC patients and cell lines is consistent the regulation of the genes by ALDH1A3 in MDA‐MB‐231 cells. In contrast, SERPINB2 was not negatively co‐expressed in all patient tumour and normal adjacent samples as expected (Fig. 1D). Together the patient tumour and cell line data prompted us to prioritize our investigation on the effects of ALDH1A3 on the plasminogen activation pathway and if this pathway contributes to ALDH1A3‐mediated invasion and metastasis in TNBC.

### 3.2 ALDH1A3 increases plasmin and ALDH1A3‐mediated invasion is plasminogen‐dependent in TNBC cells

We first confirmed that plasmin generation is altered by ALDH1A3 in TNBC cell lines by performing a cell surface plasminogen activation assay. Plasminogen is an inactive zymogen that is synthesized and secreted in the systemic circulation by the liver [36]. Plasminogen binds to cell surface receptors where it becomes cleaved by extracellular tPA and/or uPA which generates activation of the plasmin protease. In an *in vitro* plasmin activity assay, washed cell monolayers are treated with plasminogen and the subsequent generation of active plasmin is measured by hydrolysis of the synthetic substrate and the release of the chromophore pNA (chromophore).

**Fig. 2:**
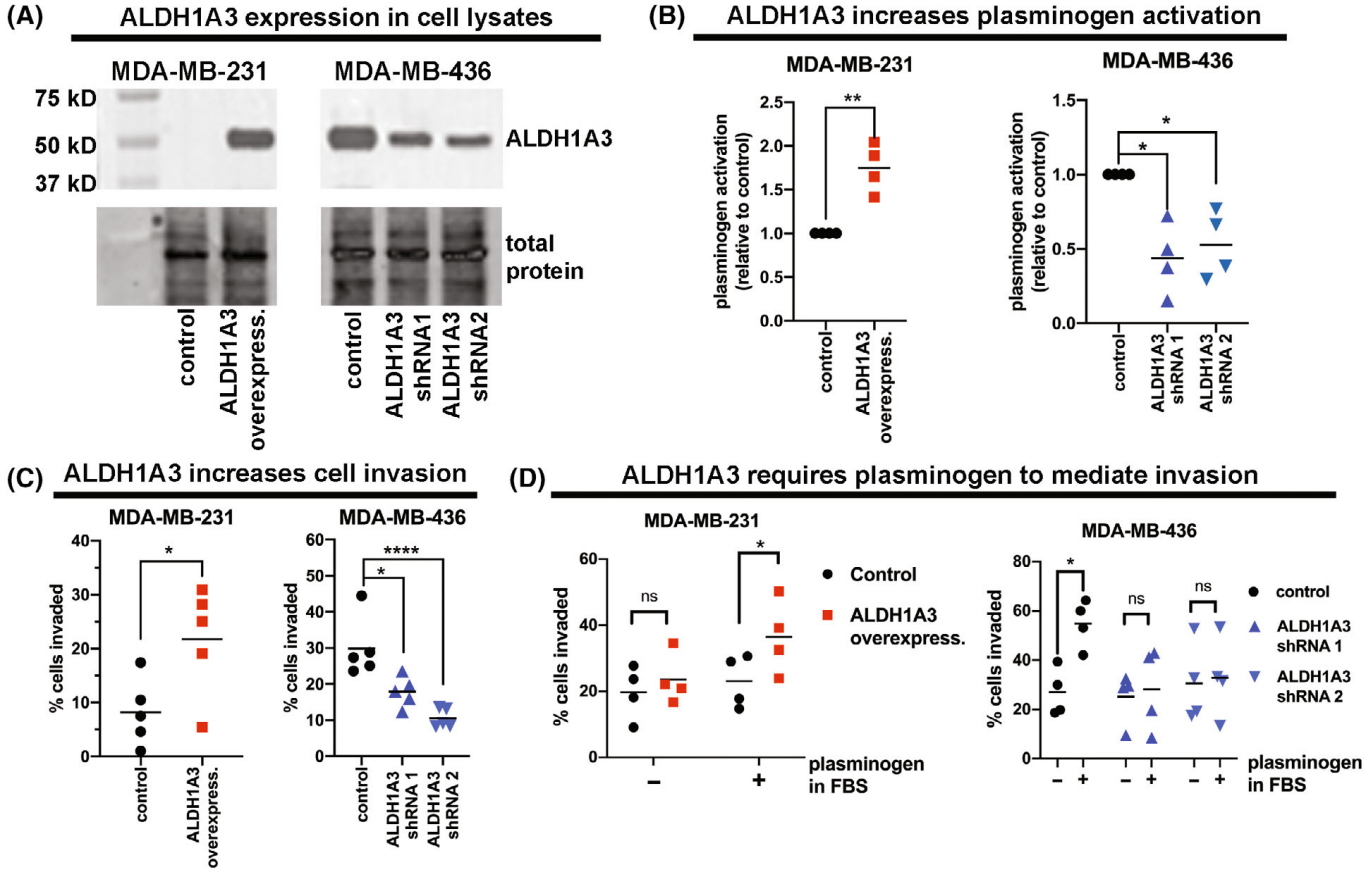
ALDH1A3 increased invasion is dependent on plasminogen activation. (A) Western blots confirmed overexpression of ALDH1A3 in MDA‐MB‐231 and reduced expression of ALDH1A3 in MDA‐MB‐436 cells. This is a representative image from three independent experiments. (B) The cell surface plasminogen activation assay was performed in MDA‐MB‐231 cells (vector control versus ALDH1A3 overexpression compared) and MDA‐MB‐436 cells (shRNA scramble control versus shRNA 1 and 2 compared). Values are relative to control cells (*n* = 4). (C, D) Transwell invasion assays were completed with MDA‐MB‐231 cells (vector control versus ALDH1A3 overexpression compared) and MDA‐MB‐436 cell (shRNA scramble control versus shRNA 1 and 2 compared) with FBS as a chemoattractant (C, *n* = 5) or with plasminogen stripped FBS and plasminogen added back as indicated (D, *n* = 5). (B–D) Significance was determined by *t*‐test for experiments with MDA‐MB‐231 cells and by one‐way ANOVA followed by multiple comparison tests for experiments with MDA‐MB‐436 cells. Significant *P* values are indicated as follows: *< 0.05, **< 0.01, ***< 0.001.

For these assays, we again used the TNBC MDA‐MB‐231 cells, with or without ALDH1A3 overexpression, as well as TNBC MDA‐MB‐436 and MDA‐MB‐468 cells, in which we knocked down ALDH1A3 (Fig. 2A, Fig. S2A). We chose this approach because ALDH1A3 is intrinsically low in MDA‐MB‐231 cells, but higher in MDA‐MB‐436 cells. We observed that the cell surface plasmin activity was positively correlated with the ALDH1A3 expression levels in these cell lines, with higher activity of plasmin in ALDH1A3 overexpressing MDA‐MB‐231 cells and lower activity upon reduction in ALDH1A3 expression by knockdown in MDA‐MB‐436 cells and MDA‐MB‐468 cells (Fig. 2B, Fig. S2B).

We next assessed the functional relevance of this activity in cancer. Protease activity is essential for the degradation of the extracellular matrix and is required for breast cancer cell invasion. We first confirmed the invasive capacity of the TNBC cell lines and noted that ALDH1A3 overexpression in MDA‐MB‐231 cells increased invasion and ALDH1A3 knockdown in MDA‐MB‐436 and MDA‐MB‐468 knockdown decreased invasion (Fig. 2C, Fig. S2C). We noted that the increase in invasion imparted by ALDH1A3 (Fig. 2C) was greater than the plasmin activity induced by ALDH1A3 (Fig. 2B). This could be due to several reasons. First, the generation of active plasmin as measured by hydrolysis of a synthetic substrate and the release of the chromophore pNA is not likely to translate equally into units of invasion as measured by a transwell assay. Invasion mediated by plasmin can also be enhanced by activation of MMPs by plasmin, hence the 1.5‐fold difference in plasminogen activity could result in amplified 3‐fold increase invasion. Repeating the transwell invasion assay with plasminogen‐depleted FBS impeded ALDH1A3‐dependent invasion, which was restored with the exogenous addition of plasminogen (Fig. 2D). Together these findings illustrate that ALDH1A3 regulates plasmin activity in TNBC, and a component of ALDH1A3‐dependent invasion is dependent on these ALDH1A3‐dependent changes in the invasion of TNBC cells.

### 3.3 ALDH1A3 increases extracellular tPA and/or uPA proteins and activity in TNBC cells

We next investigated the molecular mechanism for the regulation of plasmin activity by ALDH1A3 in TNBC cells (Fig. 2B). Using gene expression analysis of TNBC patient tumours and MDA‐MB‐231 cells, we found evidence of increased tPA and uPA, and decreased PAI‐2 (Fig. 1). PAI‐2 inactivates tPA and uPA, leading to their degradation [37]; so decreased PAI‐2 expression could also contribute to increased plasmin activity.

**Fig. 3:**
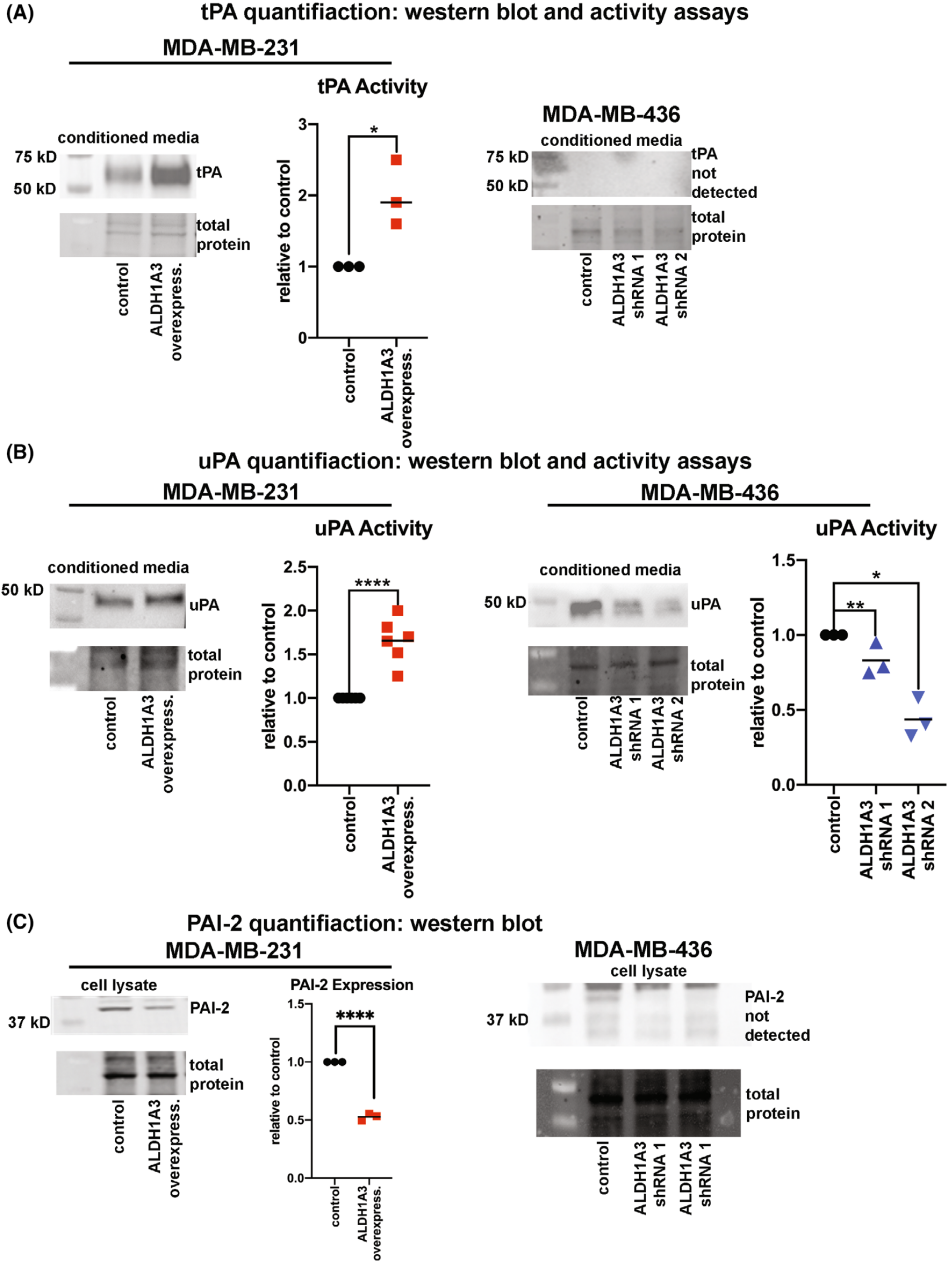
ALDH1A3 regulation of tPA, uPA, and PAI‐2 proteins and tPA and uPA activity in TNBC cells (A, B) Secreted tPA (A) and uPA (B) is detected in the CM of MDA‐MB‐231 (vector control versus ALDH1A3 overexpression compared) and MDA‐MB‐436 cells (short hairpin (shRNA) scramble control versus shRNA 1 and 2 compared) by western blots and activity assays (individual *n* are shown in the assays). tPA was not detected in CM from MDA‐MB‐436 cells. The western blot is a representative image of three independent experiment. (C) PAI‐2 is detected in the cell lysates of MDA‐MB‐231 (vector control versus ALDH1A3 overexpression compared, *n* = 3) but not detected in the cell lysates of MDA‐MB‐436 cells. (A–C) Significance was determined by *t*‐test for experiments with MDA‐MB‐231 cells and by one‐way ANOVA followed by multiple comparison tests for experiments with MDA‐MB‐436 cells. Significant *P* values are indicated as follows: *< 0.05, **< 0.01, ****< 0.0001.

We assessed the secreted tPA levels in MDA‐MB‐231, MDA‐MB‐436, MDA‐MB‐468 cells by performing western blots and tPA activity assays of the concentrated CM from the cells (Fig. 3A, Fig. S2D). In MDA‐MB‐231 and MDA‐MB‐468 cells we detected secreted tPA, which was increased upon ALDH1A3 overexpression in MDA‐MB‐231 cells (Fig. 3A) and decreased upon ALDH1A3 knockdown in MDA‐MB‐468 cells (Fig. S2D). Overexpression of ALDH1A3 in MDA‐MB‐231 also significantly increased the tPA activity (Fig. 3A) and decreased tPA activity in MDA‐MB‐468 cells (Fig. S2D). In contrast, we failed to detect secreted tPA by MDA‐MB‐436 cells.

We next assessed secreted uPA levels and activity in MDA‐MB‐231, MDA‐MB‐436 and MDA‐MB‐468 cells (Fig. 3B, Fig. S2E). ALDH1A3 increased the expression of secreted uPA in MDA‐MB‐231 cells. In MDA‐MB‐436 and MDA‐MB‐468 cells we detected secreted uPA, which was decreased by ALDH1A3 knockdown. To assess for secreted uPA activity, we performed uPA activity assays in the CM from the cells and consistent with the western blots, uPA activity was increased by ALDH1A3 overexpression in MDA‐MB‐231 cells and decreased by ALDH1A3 reduction in the MDA‐MB‐436 and MDA‐MB‐468 cells with ALDH1A3 knockdown (Fig. 3B, Fig. S2E).

We followed up on the observed SERPINB2 gene expression changes (Fig. 1) by assessing the levels of PAI‐2 in MDA‐MB‐231 and MDA‐MB‐436 cells. PAI‐2 is an intracellular protein and exerts its inhibitory activity intracellularly [38]. In MDA‐MB‐231 cells, PAI‐2 was decreased by ALDH1A3 overexpression; however, in MDA‐MB‐436 cells PAI‐2 was undetectable (Fig. 3C). In MDA‐MB‐231 cells, the decreased PAI‐2 could contribute to the increased tPA, uPA, and plasmin activity induced by ALDH1A3; however, given its absence in MDA‐MB‐436 cells, its role in ALDH1A3‐mediated plasmin activity in the breast cancer cells may be less important. Consistent with this finding in the MDA‐MB‐436 cells, the results from the patient tumour data also suggested that there was no correlation between ALDH1A3 and SERPINB2 expression (Fig. 1D). Together these data suggest that the increased plasmin activity by ALDH1A3 in TNBC could be due to both to increased secreted tPA and uPA, with some cell line‐specific effects in the dominance of one plasminogen activator over the other.

### 3.4 DNA methylation and ATRA affect PLAT expression

**Fig. 4:**
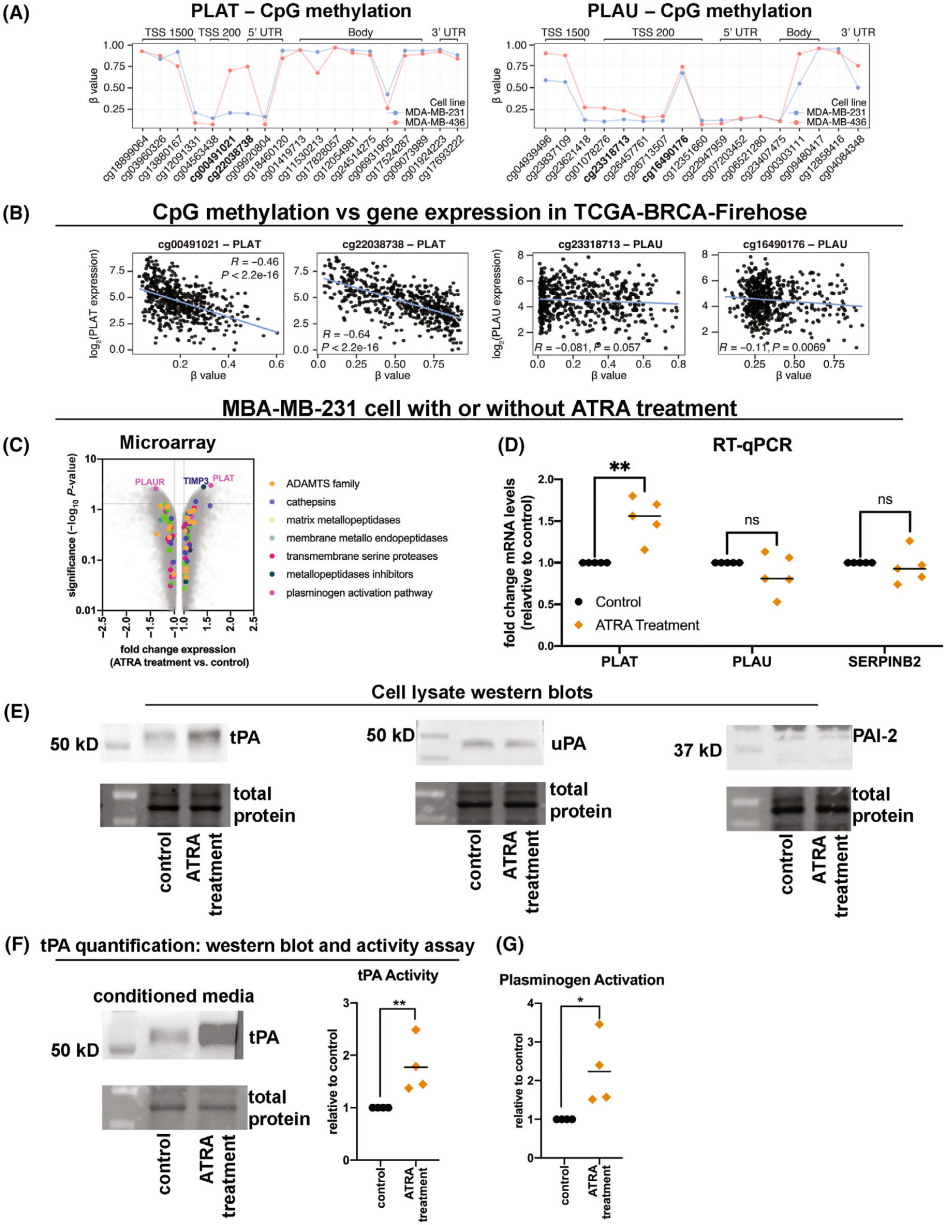
DNA methylation and ATRA affect expression of PLAT and tPA activity in TNBC cells (A) Illumina HumanMethylation450 (450K) beadchip array data of MDA‐MB‐231 and MDA‐MB‐436 cell lines were obtained from GSE78875 project accessed through the NBCI Gene Expression Omnibus (GEO). β‐values were obtained by processing the 450K data with the minfi r package. β‐values for CpG sites within the regulatory and genic regions of PLAT (plasminogen activator, tissue type, left panel) and PLAU (right panel) are shown. (B) RNA‐sequencing expression versus methylation β‐values of specific CpGs for PLAT and PLAU (plasminogen activator, urokinase) in breast cancer patient tumour samples from TCGA Firehose cohort were obtained from the Broad Institute Genome Data Analysis Centers portal. CpG sites were selected based on data available from the 450K array and genomic localization. The Spearman coefficient (*R*) and corresponding *P*‐value for the correlation between CpG methylation and gene expression are shown. (C) Transcriptome analysis of MDA‐MB‐231 cells (ATRA‐treatment versus control cells) completed by Affymetrix Human Gene 2.0ST Array (*n* = 3) identified differential expression of protease or protease regulator genes, the grey horizontal line indicates *P* < 0.05 by ANOVA method: ebayes. (D) RT‐qPCR of MDA‐MB‐231 cells (no treatment control versus 100 nm ATRA‐treated cells) assesses for effects ATRA effects on expression of PLAT, PLAU, and (serpin family B member 2, also known as plasminogen‐activator‐inhibitor 2) SERPINB2 (*n* = 5). (E) Western blots of cell lysates visually assessed for effects of ATRA treatment on tPA, uPA, and PAI‐2 in MDA‐MB‐231 cell lysates (no treatment control versus 100 nm ATRA‐treated cell). The western blot image is a representative image of three independent experiments. (F) Western blots and tPA activity assays detect secreted tPA in CM from MDA‐MB‐231 cells (no treatment control versus 100 nm ATRA‐treated cells, *n* = 4). (G) The cell surface plasminogen activation assay was performed in MDA‐MB‐231 cells (no treatment control versus 100 nm ATRA‐treated cells, *n* = 4). (D, F, G) Significance was determined by *t*‐tests and significant *P* values are indicated as follows: *< 0.05, **< 0.01 (ns, not significant).

Having demonstrated that ALDH1A3 increases plasmin activity in TNBC cells by mediating alterations in gene and protein expression of key players of the plasminogen activation pathway in the cells, we next evaluated the mechanistic basis for the activation of these genes. We were also interested in exploring potential mechanistic reasons for the lack of detectable tPA/PLAT in MDA‐MB‐436 cells (Fig. 3A). In our previous work on the mechanisms of ALDH1A3 in breast cancer, we found that DNA methylation of ALDH1A3/ATRA inducible genes plays a major role on in if a gene can be induced by ALDH1A3/ATRA. Genes with hypermethylated transcription start sites (TSS) and promoter regions are poorly inducible by ALDH1A3 [3, 39, 40]. We therefore analysed the CpG methylation of PLAT (encodes tPA) and PLAU (encodes uPA) by analysing the 450K CpG methylation data of the two genes in MDA‐MB‐231 and MDA‐MB‐436 cells (Fig. 4A). This shows that two CpG sites in the TSS are highly methylated in MDA‐MB‐436 cells and comparably unmethylated in MDA‐MB‐231 cells (CpG sites 42064880 and 42064673). Accessing the breast cancer patient tumour data from TCGA revealed that methylation of these two CpG sites is highly negatively correlated with PLAT expression (Fig. 4B). Therefore, we conclude that the lack of PLAT/tPA expression in MDA‐MB‐436 cells is due to epigenetic silencing by DNA methylation of key CpGs in the TSS. This contrasts with the expression of PLAU/uPA in both MDA‐MB‐231 and MDA‐MB‐436 cells, which is well expressed and ALDH1A3 inducible, and as shown in Fig. 4A (right), PLAU has a similar CpG methylation profile in both cell lines. Assessment of the methylation levels of a couple individual CpG methylation sites in the TSS and expression of PLAU in patient tumours did not reveal negative correlations with PLAU expression (Fig. 4B, right).

ALDH1A3's effects on breast cancer progression and gene expression have also been linked to its production of ATRA [3]. We therefore assessed for effects of ATRA on PLAT (tPA), PLAU (uPA), and SERPINB2 (PAI‐2) in MDA‐MB‐231 cells. ATRA binds to nuclear hormone retinoic acid receptors (RARs), which dimerize with retinoid‐X‐receptors (RXRs) to induce expression of genes with retinoic acid response elements (RAREs) in their promoter sequence [16]. We performed a microarray analysis of RNA extracted from MDA‐MB‐231 cells treated with 100 nm ATRA to evaluate gene expression changes in the presence of ATRA (Fig. 4C). When examining the protease and protease regulator genes, PLAT was found to be significantly increased upon ATRA treatment. RT‐qPCR confirmed increased PLAT expression upon ATRA treatment; however, unlike the gene expression effects induced by ALDH1A3 in MDA‐MB‐231 cells (Fig. 1), no significant changes were observed in SERBINB2 and PLAU (Fig. 4D). The ATRA regulation of PLAT is consistent with the observation that PLAT has a reported RARE sequence and tPA was induced by ATRA in human umbilical vein epithelial cells [41, 42].

Western blotting of cell lysates aligned with RT‐qPCR and confirmed that ATRA increased intracellular tPA but did not affect uPA or PAI‐2 levels (Fig. 4E). Consequently, ATRA treatment also increased secreted tPA and tPA activity (Fig. 4F). Cell surface plasmin activity was increased in ATRA‐treated MDA‐MB‐231 cells (Fig. 4G). Overall, these results suggest that ATRA, which is made by ALDH1A3, is sufficient to induce PLAT/tPA and increase plasmin activity; however, ATRA and ALDH1A3 are not interchangeable and ALDH1A3 has ATRA‐independent cell signalling effects not explained by ATRA production (e.g., effects on PLAU and SERPINB2 expression).

### 3.5 ALDH1A3 and tPA proteins are co‐expressed in breast cancer patient tumours

Having observed the regulation of plasmin activity and tPA/PLAT, uPA/PLAU, and PAI‐2/SERPINB2 by ALDH1A3 in cultured TNBC cells along with positive gene expression correlations between ALDH1A3 and PLAT and PLAU in TNBC patient tumours (Figs 1, 2, 3), we wondered if these correlations would be observed at the protein level in tumours.

**Fig. 5:**
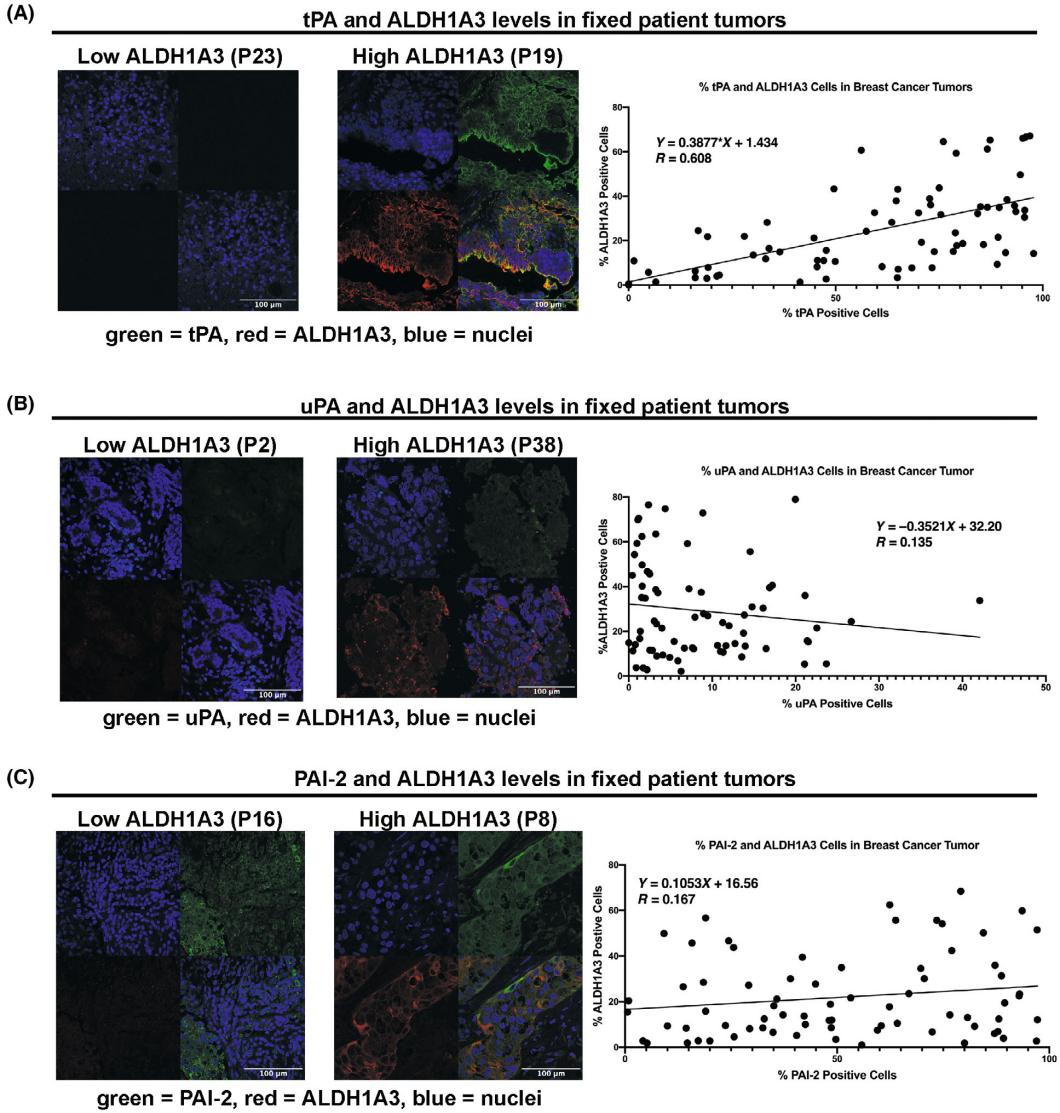
ALDH1A3 is co‐expressed with tPA but not with uPA and PAI‐2 in fixed breast cancer patient tumours. (A–C) Representative images of thin sections from a cohort of 78 formalin fixed paraffin‐embedded patient tumour samples (patient number indicated in brackets, described in Table S3) were stained with antibodies specific to ALDH1A3 (red, A–C) and tPA (green, A), uPA (green, B), PAI‐2 (green, C), and nuclei were stained with DAPI (blue) in the patient tumour samples. The scale bars = 100 μm. The graphs in A–C summarize the number of positive ALDH1A3 cells versus tPA (A), uPA (B) and PAI‐2 (C) cells quantified by imagej analysis of the stained thin sections from 73 fixed patient tumours. The number of positive cells in a patient tumour sample were based on the average numbers from random images of at least three thin sections per tumour sample. Linear regression analysis of the graphs determined the co‐expression correlation based on the slope and *R* value.

We assessed a cohort of 78 archived fixed primary, treatment naïve, human breast tumour samples for co‐expression between ALDH1A3 and tPA, uPA, and PAI‐2 (Table S3, tumour pathology and clinical details summarized). We include representative images of patient tumour sections stained with ALDH1A3 and tPA (Fig. 5A), ALDH1A3 and uPA (Fig. 5B), and ALDH1A3 and PAI‐2 (Fig. 5C) and include examples of patient tumour samples that had low and high ALDH1A3 staining. Analysis of the patient samples revealed that ALDH1A3 staining was significantly and positively correlated with tPA staining, where ALDH1A3 and tPA were often co‐expressed in the same cells (Fig. 5A). In contrast, ALDH1A3 staining was not positively correlated with uPA staining (Fig. 5B), nor negatively correlated with PAI‐2 (Fig. 5C) as hypothesized. This data suggests that the *in vitro* regulation of plasminogen activation by ALDH1A3 is strongly reflected between ALDH1A3 and tPA in breast cancer patient tumours but not apparent between ALDH1A3 and uPA, or ALDH1A3 and PAI‐2.

### 3.6 ALDH1A3 and tPA proteins are co‐expressed in patient tumour cells and associated with TNBC subtype, high tumour grade, and worse progression‐free survival

**Fig. 6:**
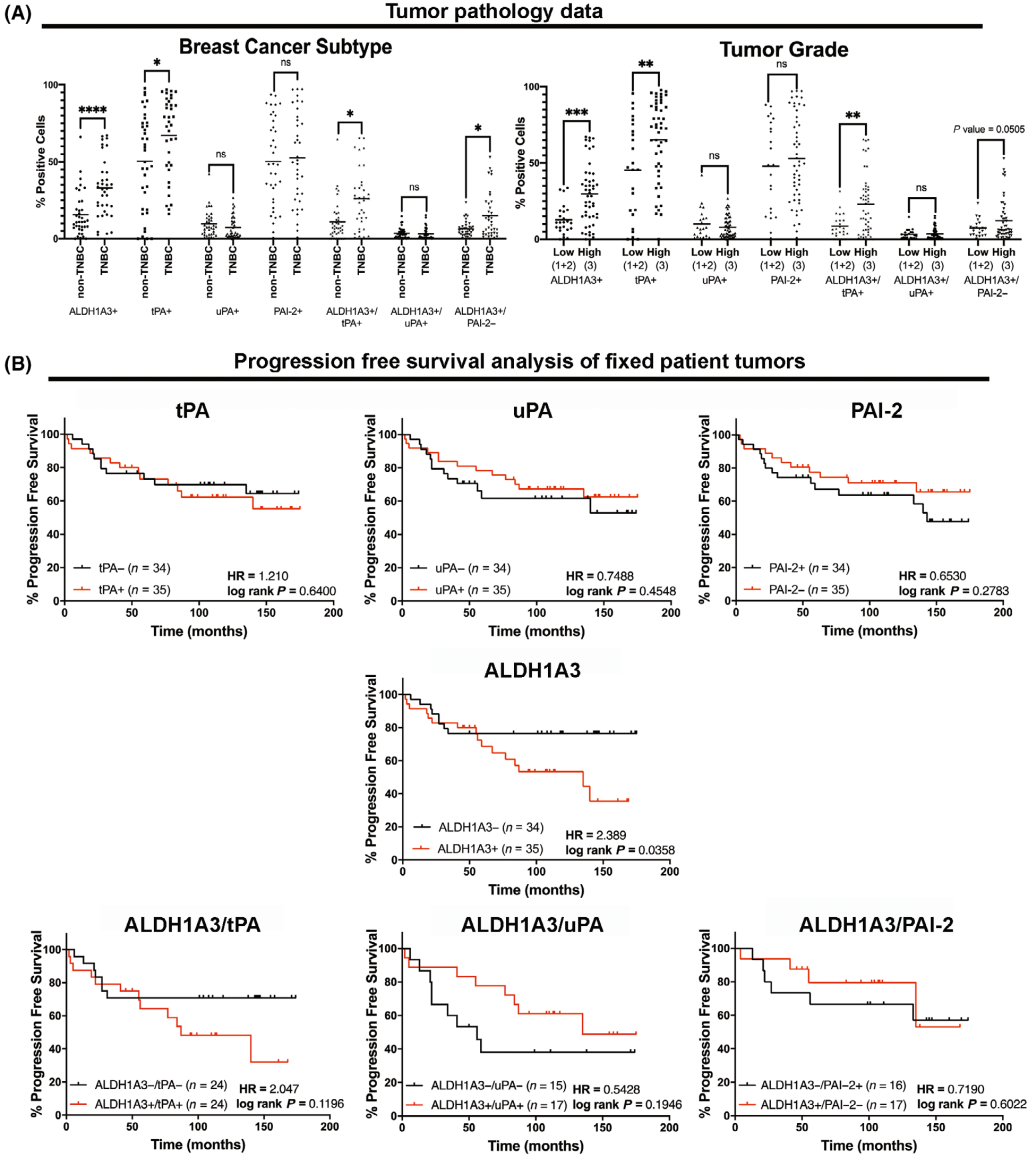
ALDH1A3 and tPA protein levels, but not uPA and PAI‐2, are associated with the TNBC subtype, high tumour grade, and worse progression‐free survival. (A) The panel of 78 formalin‐fixed paraffin‐embedded patient tumour samples (details of patient samples are provided in Table S3) are stained for ALDH1A3, tPA, uPA, and PAI‐2 in the tumour samples in Fig. 5 and quantified for positive and negative cells were divided into two groups based on breast cancer subtype (top left graph, non‐TNBC = hormone receptor positive and HER+) and tumour grade (top right graph, low = grade 1 and 2, high = grade 3). Significance was determined by *t*‐tests and significant *P* values are indicated as follows: *< 0.05, **< 0.01, ***< 0.001, ****< 0.0001; ns, not significant. (B) Kaplan–Meier progression‐free survival analysis of the 73 breast cancer patient tumour cohort based on median number of ALDH1A3, tPA, uPA and PAI‐2 positive cells or ALDH1A3 in combination with tPA, uPA, and PAI‐2. HR, hazard ratio and significance determined by the log‐rank test of survival probability is indicated as log rank *P*.

We analysed for possible correlations between tumour pathology parameters and progression‐free survival based on the percentage of positive ALDH1A3, tPA, uPA, and PAI‐2 cells, or the combination of ALDH1A3 with tPA or uPA, or ALDH1A3 combined with PAI‐2 negative cells. We assessed for associations with tumour stage, tumour grade, subtype, and lymph node involvement. We noted significant associations between high numbers of ALDH1A3+ and tPA+ cells and the TNBC subtype (non‐TNBC versus TNBC) and higher tumour grade (grade 1 and 2 versus grade 3, Fig. 6A). We did not observe any significant correlations with other tumour pathology parameters (Fig. S3A,B; TMN stage and lymph node involvement). Whether we segregated patients based on individual lymph node involvement (yes/no), or lymph node stage (N0, N1, N2, N3) we did not obtain any significant correlations with ALDH1A3, tPA or uPA (Fig. S3C). The few patient tumour samples that had high levels of lymph node involvement (i.e., N2 or N3) limited the power of the analysis. Although we did not detect significant correlations, there were some trends with patients in the N2 or N3 groups having higher levels of tPA and uPA staining.

Next, we determined if having high (top 50% of tumours) versus low (bottom 50% of tumours) percentages of positive ALDH1A3, tPA, uPA, and PAI‐2 cells, or having combined high ALDH1A3 and tPA (ALDH1A3+/tPA+) versus low ALDH1A3−/tPA−, high ALDH1A3 and uPA (ALDH1A3+/uPA+) versus low ALDH1A3−/uPA−, high ALDH1A3 and low PAI‐2 (ALDH1A3+/PAI‐2−) versus low ALDH1A3 and high PAI‐2 (ALDH1A3−/PAI‐2+) cells in patient tumours is associated with later disease progression. This revealed that ALDH1A3+ cells (HR = 2.389, log rank *P* = 0.0358) had the strongest association with disease progression, followed by the combination of ALDH1A3+/tPA+ cells (HR = 2.047, log rank *P* = 0.1196), and tPA+ alone (HR = 1.210, log rank *P* = 0.64), while uPA+ and PAI‐2− was weakly associated disease‐free progression (trend, not significant) (Fig. 6B). Pairing ALDH1A3 with uPA or PAI‐2 abrogated the association of ALDH1A3 with progression.

Overall, the staining of fixed breast cancer patient tumours suggests that among these four proteins, ALDH1A3 has the strongest associations with the TNBC subtype, higher tumour grade, and later recurrence (progression) and that tPA has similar associations that overlap with ALDH1A3. The lack of increased significant correlation with worse progression survival when we assessed the combination of ALDH1A3+/tPA+ cells could be due the reduced number of patients analysed when a double positive/double negative analysis was performed (total patients = 48, Fig. 6B) versus the greater patients when the single ALDH1A3+ stain analysis was performed (total patients = 69, Fig. 6B). The progression‐free survival analysis also suggests that tPA alone is not a strong predictor of worse progression free survival and that having tPA alone is not sufficient to promote later metastasis/recurrence. ALDH1A3 effects on later metastasis development suggested by strong associations with worse progression free survival as likely multifactorial and include other factors induced by ALDH1A3, beyond tPA. Regardless, the observed significant co‐expression of ALDH1A3 with tPA (Fig. 6A) suggests that when ALDH1A3 is expressed in a patient tumour, tPA is most likely present.

### 3.7 tPA mediates plasmin activity, invasion, and increases lymph node metastasis of MDA‐MB‐231 cells

**Fig. 7:**
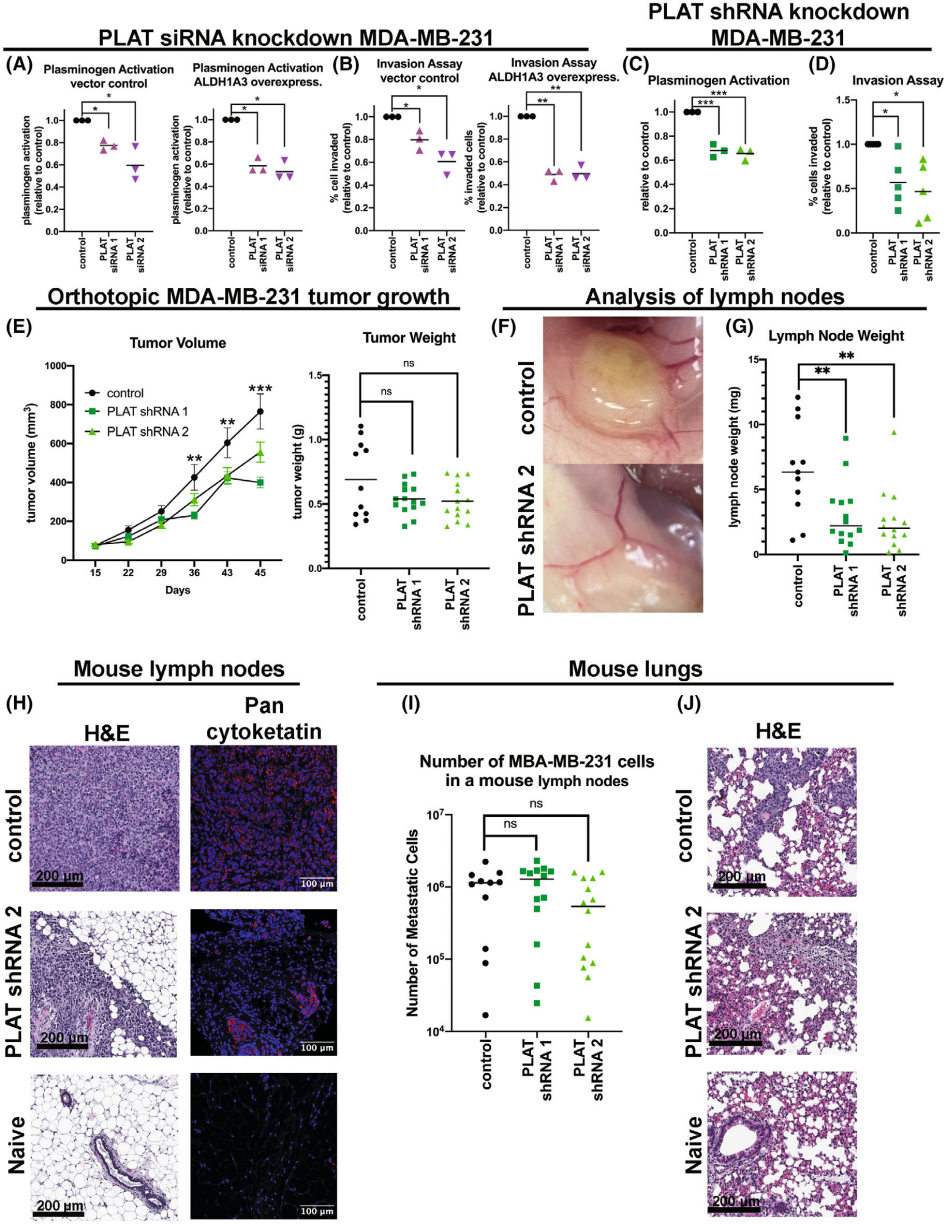
tPA knockdown reduces plasmin and invasion mediated by ALDH1A3 and lymph node metastasis of MDA‐MB‐231 cells orthotopically implanted in NOD/SCID mice. (A, B) The cell surface plasminogen activation assay (A) or transwell invasion assay (B) was performed in MDA‐MB‐231 cells with or without ALDH1A3 overexpression treated with plasminogen activator, tissue type (PLAT) small interfering RNA (siRNA) 1 and 2. (C, D) The cell surface plasminogen activation assay (C) or transwell invasion assay (D) was performed in MDA‐MB‐231 cells with short hairpin (shRNA) PLAT knockdown. (A–D) Significance determined by one‐way ANOVA followed by multiple comparison and significant *P* values are indicated as follows: *< 0.05, **< 0.01, ***< 0.001. (E) Tumour volume and weights of mice injected with MDA‐MB‐231 shRNA scramble control, PLAT shRNA 1, and PLAT shRNA 2. Significance was determined through a one‐way ANOVA followed by multiple comparison (*n* = 11, 15 and 15, respectively) and significant *P* values are indicated as follows: **< 0.01, ***< 0.001; ns, not significant. (F, G) Representative images (F) and total lymph node weights (G, axillary and inguinal nodes combined per mouse) of mice from E. Significance was determined through a one‐way ANOVA followed by multiple comparison (*n* = 11, 15 and 15 respectively) and significant *P* values are indicated as follows: **< 0.01. (H) Representative cropped images of H&E‐stained lymph node sections (scale bar = 200 μm, 10× zoom of the scanned image of full lymph node sections, Fig. S5) and pan cytokeratin‐stained slides of the lymph nodes (scale bar = 100 μm, *n* = 4 for control, 4 for PLAT shRNA 2, and 1 for Naïve). (I, J) Analysis of mouse lungs from E. (I) Quantification of MDA‐MB‐231 cells (control versus shRNA 1 or 2) present in lung lobes of each mouse by real time‐quantitative polymerase chain reaction using human‐specific glyceraldehyde 3‐phosphate dehydrogenase (GAPDH) primers (the horizontal line indicates the median). Analysis for significance was determined by a one‐way ANOVA, followed by multiple comparisons (*n* = 11, 15 and 15, respectively), and the results suggest there is no significant difference as indicated by ns (not significant). (J) Representative cropped images of H&E‐stained lung lobe sections (scale bar = 200 μm, 10× zoom of scanned image of full lung lobe sections, Fig. S5, *n* = 4 for control, 4 for PLAT shRNA 2, and 1 for Naïve).

Having shown that in TNBC, ALDH1A3 regulates the plasminogen activation pathway, depends upon plasminogen for invasion, and among the plasminogen activation pathway factors is most strongly associated with PLAT/tPA, we next wondered if tPA affects plasmin activity, invasion, and TNBC tumour growth and metastasis. We, therefore, knocked down PLAT in MDA‐MB‐231 cells, with or without ALDH1A3 overexpression, by transient siRNA expression confirmed that this reduced PLAT/tPA and tPA activity in the TNBC cells (Fig. S4A). SiRNA Knockdown of PLAT/tPA reduced plasmin generation (Fig. 7A) and invasion (Fig. 7B) in vector control and ALDH1A3 overexpressing cells.

To examine the relationship between tPA and tumour progression *in vivo*, we generated stable knockdown of PLAT/tPA in MDA‐MB‐231 cells and these cells has reduced tPA activity (Fig. S4B) and had reduced plasmin and invasion activity (Fig. 7C,D). We orthotopically implanted these MDA‐MB‐231 cells in immunocompromised female NOD/SCID mice and measured tumour growth and metastasis to the lymph nodes and lungs. Knockdown of tPA resulted in a significant decrease in tumour volume; however, at termination, the harvested tumours were not significantly smaller (Fig. 7E). We examined the mice at termination and noted visual differences in the axillary and inguinal lymph nodes, where some mice had noticeably enlarged axillary and/or inguinal lymph nodes indicative of metastatic disease (Fig. 7F). We collected the axillary and inguinal lymph nodes from each mouse and weighed the lymph nodes. This revealed a significantly lower total lymph node weight in mice that had been implanted with MDA‐MB‐231 cells with reduced tPA expression by knockdown (Fig. 7G). We confirmed that the enlarged lymph nodes consisted of predominately metastatic MDA‐MB‐231 cells by staining the fixed lymph nodes sections by H&E and anti‐pan‐cytokeratin antibody (stains epithelial cell specifically, Fig. 7H, Fig. S5A, full images of node sections). As a negative control, we include analysis of lymph nodes harvested from a naïve mouse that had not been implanted with MDA‐MB‐231 cells (bottom images, Fig. 7H). The epithelial cells were absent in the lymph nodes of the negative control naïve mouse.

Finally, we examined the lungs for metastasis. We quantified the number of disseminated MDA‐MB‐231 cells using an RT‐qPCR‐based method which can accurately quantify between 10^2^ and 10^6^ MDA‐MB‐231 cells in the lung lobe of a mouse [31]. This revealed evidence of lung metastasis (Fig. 7I), which we confirmed by H&E (Fig. 7J, Fig. S5B, full images of lung lobe sections). Although not significant, we observed a trend in reduced cancer cells in the lungs of mice implanted with MDA‐MB‐231 cells with reduced tPA expression by knockdown (Fig. 7I). Together these analyses suggest that reduced tPA in MDA‐MB‐231 cells impedes the early stage of metastatic dissemination (i.e., to the lymph nodes); however, tPA reduction alone is not sufficient to significantly reduce overall metastasis as seen in the analysis of the lungs.

## 4 Discussion

ALDH1A3 has been shown to correlate with poor patient survival, disease progression, and recurrence in many cancers, including breast, prostate, glioblastoma, neuroblastoma, pancreatic, gastric, gall bladder, colon, and intrahepatic cholangiocarcinoma cancers [2, 3, 14, 43, 44, 45, 46, 47, 48, 49]. Investigations into the function of ALDH1A3 in cancer suggest it promotes disease progression by both increasing tumour burden and metastasis [3, 4, 5, 43]. Given that metastasis is the primary cause of cancer mortality it is critical to characterize pathways and factors that promote metastasis and therefore focus our investigation on understanding ALDH1A3‐mediated invasion and metastasis. Previous studies have linked ALDH1A3‐mediated cancer progression to gene expression changes and ATRA [3, 5, 13], effects on epithelial‐mesenchymal‐transition [3, 10, 14], and altered glucose and gamma‐aminobutyric acid metabolism [7, 8], however, the specific factors that mediate ALDH1A3 invasion and metastasis are largely unidentified. We, therefore, performed analyses to specifically identify factors that mediate the early stage of metastasis; the proteases and regulators of proteases that mediate cancer cell invasion through the remodelling of the extracellular matrix.

Our analyses of TNBC show that ALDH1A3 transcriptionally regulates the plasminogen activation pathway, resulting in increased activity of the serine protease plasmin, which we link to ALDH1A3‐mediated invasion. Specifically, in TNBC cells we find that ALDH1A3 can transcriptionally regulate PLAT, PLAU, and SERPINB2; however, considering both the cell line and patient tumour data, the strongest overall evidence was between ALDH1A3 and PLAT/tPA. In patient tumours, co‐expression of ALDH1A3 and tPA was strongly correlated and we noted a positive association with the TNBC subtype, higher grade tumours, and worse progression‐free survival.

It is noteworthy that in our gene expression analyses of PLAT, PLAU, and SERPINB2, we also found that PLAT was inducible by the nuclear hormone receptor ligand ATRA but PLAU and SERPINB2 were not. Notably, among PLAT, PLAU, and SERPINB2, only PLAT has been described to have a RARE, which is inducible ATRA [41, 42]. Uchida et al., also showed that ATRA increased tPA activity and *in vitro* invasion in human oral squamous‐cell‐carcinoma line [50]. Considering our current data showing that tPA promotes metastasis of MDA‐MB‐231 cells and is inducible by ATRA, it also partly explains our prior findings where like ALDH1A3, ATRA increased metastasis of MDA‐MB‐231 tumours [3]. In contrast to ATRA‐inducible PLAT/tPA, other ALDH1A3‐downstream co‐regulatory mechanisms could be at play in the regulation of at least PLAU and SERPINB2, beyond the production of ATRA by ALDH1A3. In this context, ATRA‐mediated induction of uPA in endothelial cells absent a RARE sequence has been described, where ATRA induces expression of RARs and RAR:RXRs heterodimers interact with Sp1 which ultimately leads to the transcription of uPA [51]. This reveals the dependence on other factors, which may be cell line or patient tumour‐specific factors that may be less commonly expressed. For example, it has been previously described that ALDH1A3 regulates gene expression via microRNAs [47], long non‐coding RNAs [52, 53, 54, 55, 56, 57], and activation of the phosphatidylinositol 3‐kinase/Protein kinase B/rapamycin (PI3K/AKT/mTOR) signalling pathway [58]. It is possible that these ALDH1A3‐regulated factors are contributing to the regulation of PLAU and SERPINB2 by ALDH1A3 and ATRA does not fully replicate the cell signalling events induced by ALDH1A3.

The plasminogen activator tPA plays a physiologically important role in fibrinolysis and clot dissolution due to its function in plasminogen activation [59]. Several studies have also suggested a potential role of tPA in cancer progression based on expression and associations in patient tumours and blood samples. In 2005, Corte et al. [60], performed ELISA assays on homogenized tumour extracts from breast cancer patients to quantify cytosolic tumour tPA levels and noted that in only the subgroup of patients with lymph node‐negative disease, tPA was associated with better overall survival; no correlations were found in other patient subgroups. Other studies have investigated associations between cancer progression and serum levels of tPA (not in tumours). For example, low plasma/serum level of tPA was associated with poor disease‐free survival and enhanced risk of breast cancer progression [61]. While, in other studies, higher levels of plasma tPA are linked to a greater risk of breast cancer and aggressive disease [62, 63, 64]. Although the above‐mentioned studies indicate tPA as a biomarker of progressive (or non‐progressive) breast cancer, an evaluation of the functional role of the protein upon knockdown or overexpression in cancer cells was lacking. Our current study is the first one to examine the function of tPA expressed by cancer cells in tumour growth and metastasis using an orthotopic xenograft breast tumour mouse model. Although tPA knockdown did not significantly reduce lung metastatic burden, it did reduce lymph node metastatic burden. Several factors could contribute the lack of consistency between lymph node involvement in the patients and tPA KD observed in the mouse model employed here. First, the patient tumours are more complex and heterogenous, which can differentially influence the lymph node involvement, including the expediated timeline form initial tumour formation in the mouse model to metastasis, which occurs in a matter of weeks in the tumour xenograft model, but could take years in humans. Furthermore, the lack of a functional adaptive immune system in our mouse tumour mode could alter the metastatic trajectory of the cells in comparison to humans. Overall, these data suggest tPA contributes to the metastatic trajectory of breast cancer cells but tPA reduction is not sufficient to inhibit lung metastasis. Notably, plasmin activation is mediated by multiple factors in addition to tPA (e.g., uPA); therefore, tPA knockdown alone only partially reduces plasmin activity. Residual plasmin activity generated by uPA could be sufficient to mediate lung metastasis despite the reduced lymph node metastasis we observed.

In exploring to potential relationships between ALDH1A3 and uPA and PAI‐2 in breast cancer patients, we also performed individual analyses of these proteins in fixed breast cancer patient tumour samples. Although we did not note significant correlations here with uPA, others have shown that high blood uPA is an independent predictor of metastatic breast cancer progression [65]. PAI‐2 has been reported to be associated with progression‐free survival and good outcomes [66]; we report a similar trend that is consistent with those previous reports. The lack of significance in our study could be explained by the smaller patient cohort we assessed. Finally, we acknowledge that breast cancer is a complex, heterogenous disease, where tumour–host interface plays an important role in cancer progression [67]. Single gene knockdown studies in mouse models can only provide a limited understanding of the complex multidimensional disease of cancer. Future studies with ALDH1A3 and tPA‐plasmin axis link will/should investigate in the context of the complexity of the tumour ecological system.

## 5 Conclusions

In summary, our analyses suggest a novel mechanism of ALDH1A3‐mediated invasion and metastasis in TNBC via the regulation of the plasminogen activation pathway. This pathway has multiple players and levels of regulation, and our evidence strongly links ALDH1A3 with tPA in TNBC. ALDH1A3 is an important player in the progression of several other cancers, hence it will be crucial to evaluate if ALDH1A3 regulates the plasminogen activation pathway in these cancers as well. It is also clear from our analyses and the review of the literature that ALDH1A3 has multi‐factorial effects in cancer progression which is unlikely to be explained by a single gene or protein. Therefore, strategies that target ALDH1A3 specifically may remain the best way to ensure therapeutic effects are broadly applicable.

## Conflict of interest

The authors declare no conflict of interest.

## Author contributions

AGB, MEM, MLD, M‐CDW, PM designed the study and performed the experiments, performed data analysis, and data interpretation and wrote the manuscript. HFC, RPA, OLW, BMC, WF, and JV performed the experiments, and interpreted the data. PJB, GB, GK, LKH, and CAG performed study design, and data acquisition, analysis, and interpretation. PM conceptualized and supervised the study and acquired the funding. DMW acquired the funding, performed data interpretation and analysis, and supervised the study. All authors edited and revised the manuscript and were involved in the final approval of the manuscript.

### Peer review

The peer review history for this article is available at https://www.webofscience.com/api/gateway/wos/peer‐review/10.1002/1878‐0261.13528.

## Supporting information


**Fig. S1.** ALDH1A3 expression correlations with PLAU, PLAT and SERPINB2 in HR+ breast cancer patient tumours, normal adjacent tissues, TNBC cell lines and HER2+ breast cancer patient tumours.
**Fig. S2.** Knockdown of ALDH1A3 in MDA‐MB‐468 cells reduces plasmin, tPA and uPA activity.
**Fig. S3.** ALDH1A3, tPA, uPA and PAI‐2 do not correlate with tumour stage or lymph node involvement.
**Fig. S4.** PLAT knockdown in MDA‐MB‐231 cells is confirmed by western blotting and tPA activity assays.
**Fig. S5.** The full scanned images of the cropped images from Fig. 7.
**Table S1.** shRNA and siRNA sequences and clones.
**Table S2.** Gene‐specific primers used in RT‐qPCR.
**Table S3.** Summary of Patient Tumor Pathology and Clinical Data.
**Table S4.** Families of Proteases or Regulators of Proteases Genes Implicated in the Remodeling of the Extracellular Matix.

## Data Availability

Supplemental Figures and Tables are available in the Supporting Information. Gene expression data for MDA‐MB‐231 control and overexpressing ALDH1A3 is available at GSE103426 at the Gene Expression Omnibus (GEO). The 450K methylation data is available at GSE78875 at the GEO. TCGA patient tumour data can be accessed at the cbioportal site and the Broad Institute GDAC portal.
