# Supplementary material for: ALDH1A3 promotes invasion and metastasis in triple‐negative breast cancer by regulating the plasminogen activation pathway
Source: Mol Oncol. 2023 Oct 9;18(1):91–112. doi: 10.1002/1878-0261.13528 (PMC10766202; doi:10.1002/1878-0261.13528)
Supplement: Supplementary file 1 — Fig. S1. ALDH1A3 expression correlations with PLAU, PLAT and SERPINB2 in HR+ breast cancer patient tumours, normal adjacent tissues, TNBC cell lines and HER2+ breast cancer patient tumours. Fig. S2. Knockdown of ALDH1A3 in MDA‐MB‐468 cells reduces plasmin, tPA and uPA activity. Fig. S3. ALDH1A3, tPA, uPA and PAI‐2 do not correlate with tumour stage or lymph node involvement. Fig. S4. PLAT knockdown in MDA‐MB‐231 cells is confirmed by western blotting and tPA activity assays. Fig. S5. The full scanned images of the cropped images from Fig. 7. Table S1. shRNA and siRNA sequences and clones. Table S2. Gene‐specific primers used in RT‐qPCR. Table S3. Summary of Patient Tumor Pathology and Clinical Data. Table S4. Families of Proteases or Regulators of Proteases Genes Implicated in the Remodeling of the Extracellular Matix. [file MOL2-18-91-s001.docx]

**SUPPLEMENTAL TABLES**

**Supplemental Table S1. shRNA and siRNA sequences and clones**

| **Gene** | **shRNA** | **Sequence** |
| --- | --- | --- |
| ALDH1A3 | shRNA1 | TGCTGTTGACAGTGAGCGCGCATAGCAAATCCTAGGATAA |
|  | shRNA2 | TAGTGAAGCCACAGATGTATTATCCTAGGATTTGCTATGCT |
|  |  |  |
|  | |  |
| **Gene** | **shRNA** | **shRNA Used** |
| PLAT | shRNA1 | V3LHS_399215 |
|  | shRNA2 | V2LHS_11084 |
|  | |  |
| **Gene** | **siRNA** | **siRNA Used** |
| PLAT | siRNA1 | AAGUGUCAUCAUCGAAUU |
|  | siRNA2 | UUCUGUUAAGUAAAUGUU |

**Supplemental Table S2. Gene-specific primers used in RT-qPCR**

| **Target Gene** | **Forward primer sequence** | **Reverse primer sequence** |
| --- | --- | --- |
| B2M | AGGCTATCCAGCGTACTCCA | CGGATGGATGAAACCCAGACA |
| ARF1 | GTGTTCGCCAACAAGCAGG | CAGTTCCTGTGGCGTAGTGA |
| PUM1 | GGCGTTAGCATGGTGGAGTA | CATCCCTTGGGCCAAATCCT |
| ALDH1A3 | TCTCGACAAAGCCCTGAAGT | TATTCGGCCAAAGCGTATTC |
| PLAT | TGTGTGGAGCAGTCTTCGTT | TCGCTGCAACCTTGGTAAGA |
| PLAU | GTCACCTACGTGTGTGGAGG | AGTTAAGCCTTGAGCGACCC |
| SERPINB2 | GCAGTTACCCCCATGACTCC | GTGCCTGCAAAATCGCATCA |
| GAPDH (human) | CAAGGCTGAGAACGGGAAG | GCGAGACCCCACTAACATCA |
| GAPDH (mouse) | CGCCCCACTTGATTTTGGAG | GGCGGAGATGATGACCCTTT |

**Supplemental Table S3. Summary of Patient Tumor Pathology and Clinical Data**

| **Patient ID** | **Type** | **Subtype** | **Staging** | **Cancer Stage** | **Tumor Grade** | **Lymph node Involvement** | **Progression Free?** | **Study** |
| --- | --- | --- | --- | --- | --- | --- | --- | --- |
| P1 | IDC | ER+/PR+ | T2N1M0 | IIB | 2 | yes | yes | 1,2,3 |
| P2 | IDC | ER+/PR+/HER2+ | T1N1M0 | IIA | 2 | yes | yes | 1,2,3 |
| P3 | IDC | TNBC | T1N3M0 | IIIC | 3 | yes | yes | 1,2,3 |
| P4 | IDC | TNBC | T4N1M0 | IIIB | 3 | yes | yes | 1,2,3 |
| P5 | ILC | ER+/PR+/HER2+ | T3N3M0 | IIIB | 3 | yes | no | 1,2,3 |
| P6 | IDC | ER+/PR+/HER2+ | T2N3M0 | IIIC | 3 | yes | yes | 1,2,3 |
| P7 | IDC | ER+/PR+ | T1N0M0 | I | 2 | no | no | 1,2,3 |
| P8 | IDC | TNBC | T1N0M0 | I | 3 | no | yes | 1,2,3 |
| P9 | IDC | ER+/PR+/HER2+ | T4N3M0 | IIIC | 3 | yes | no | 1,2,3 |
| P10 | IDC | ER+/PR+ | T2N0M0 | IIA | 1 | no | yes | 1,2,3 |
| P11 | IDC | ER+/PR+/HER2+ | T2N0M0 | IIA | 3 | no | no | 1,2,3 |
| P12 | IDC | ER+/PR+ | T2N0M0 | IIA | 3 | no | no | 1,2,3 |
| P13 | IDC | ER+/PR+ | T2N0 | IIA | 3 | no | no | 1,2,3 |
| P14 | IDC | TNBC | T2N1M0 | IIB | 3 | yes | no | 1,2,3 |
| P15 | IDC | ER+/PR+ | T2N1M0 | IIB | 2 | yes | no | 1,2,3 |
| P16 | IDC | ER+/PR+/HER2+ | T1N1M0 | IIA | 3 | yes | no | 1,2 |
| P17 | ILC | ER+/PR+ | T2N1M0 | IIB | 2 | yes | yes | 1,3 |
| P18 | IDC | ER+/PR+ | T2N1M0 | IIB | 3 | yes | no | 1,2,3 |
| P19 | IDC | HER2+ | T2N0M0 | IIA | 3 | no | yes | 1,2,3 |
| P20 | IDC | ER+/PR+ | T1N2M0 | IIIA | 3 | yes | yes | 1,2,3 |
| P21 | IDC and ILC | ER+/PR+ | T2N0MO | IIA | 2 | no | yes | 1,2 |
| P22 | IDC | ER+ | T1N0M0 | I | 1 | no | no | 1,2,3 |
| P23 | IDC | ER+/PR+ | T2N0M0 | IIA | 2 | no | yes | 1,2,3 |
| P24 | IDC | ND | T1micN0 | I | 3 | no | yes | 1,2,3 |
| P25 | ILC and IDC | ER+/PR+ | T2N0 | IIA | 2 | no | yes | 1,2,3 |
| P26 | IDC | ER+/HER2 | T1cN1M0 | IIA | 3 | yes | yes | 1,2,3 |
| P27 | IDC | ER+/PR+ | T2N1a | IV | 2 | yes | yes | 1,2,3 |
| P28 | IDC | ER+/PR+ | T2N0M0 | IIA | 3 | no | no | 1,2,3 |
| P29 | IDC | ER+/PR+/HER2+ | T1cN0M0 | I | 2 | no | yes | 1,2,3 |
| P30 | IDC | ER+/PR+/HER2+ | T1cN0M0 | I | 2 | no | yes | 1,3 |
| P31 | IDC | ER+/PR+ | T2N0M0 | IIA | 2 | no | no | 1,2,3 |
| P32 | IDC | TNBC | T2N1M0 | IIB | 3 | yes | yes | 1,2,3 |
| P33 | ILC | ER+/PR+ | T2N1 | IIB | 2 | yes | yes | 1,2,3 |
| P34 | IDC | ER+/PR+ | T2N2aM0 | IIIA | 1 | yes | yes | 1,2,3 |
| P35 | IDC | ER+/PR+ | T2N2aM0 | IIIA | 1 | yes | yes | 1 |
| P36 | IDC | ER+/PR+ | T3 | IIIB | 2 | yes | yes | 1,2,3 |
| P37 | IDC | TNBC | T2,N0 | IIA | 3 | no | yes | 1,2,3 |
| P38 | IDC | TNBC | T2N2 | IIIa | 3 | yes | yes | 1,2,3 |
| P39 | IDC | ER+/PR+ | T2N0 | IIA | 2 | no | no | 1,2,3 |
| P40 | IDC | TNBC | T1N0 | I | 3 | no | yes | 1,2,3 |
| P41 | IDC | ER+ | T2N2 | IIIA | 2 | yes | yes | 1,2,3 |
| P42 | ILC | ER+/PR+ | T1b | I | 2 | no | yes | 1,2,3 |
| P43 | IDC | ER+/PR+/HER2+ | T2N1 | IIB | 3 | yes | no | 1,2,3 |
| P44 | IDC | ER+/PR+ | T1N0 | I | 2 | no | yes | 1,2,3 |
| P45 | ILC | ER+/PR+ | T1 | I | 3 | no | no | 1,2 |
| P46 | IDC | TNBC | T2N0 | IIA | 3 | no | no | 1,2,3 |
| P47 | IDC | ER+/PR+/HER2+ | T1N0 | I | 3 | no | yes | 1,2,3 |
| P48 | IDC | TNBC | T1N0 | I | 3 | no | no | 1,2,3 |
| P49 | DCIS | TNBC | T4 N1 M0 | IIIB | 3 | Yes | yes | 1,2,3 |
| P50 | DCIS | TNBC | Tis N0 N0 | I | 2 | No | yes | 1,2,3 |
| P51 | DCIS and IDC | TNBC | T1 N0 M0 | I | 3 | No | yes | 1,2,3 |
| P52 | IDC | TNBC | T4 N0 M0 | IIIB | 3 | No | yes | 1,2,3 |
| P53 | DCIS and IDC | TNBC | T1 N1 M0 | IIA | 3 | Yes | yes | 1,2,3 |
| P54 | DCIS and IDC | TNBC | T1 N0 M0 | I | 2 | No | no | 1,2,3 |
| P55 | DCIS and IDC | TNBC | T2 N0 M0 | IIA | 3 | Yes | yes | 1,2,3 |
| P56 | DCIS and IDC | TNBC | T2 N1 M0 | IIB | 3 | Yes | yes | 1,2,3 |
| P57 | DCIS and IDC | TNBC | T1 N0 M0 | I | 3 | No | yes | 1,2,3 |
| P58 | IDC | TNBC | T2 N1 M0 | IIB | 3 | yes | no | 1,2,3 |
| P59 | IDC | TNBC | T3 N2 M0 | IIIA | 3 | yes | no | 1,2,3 |
| P60 | IDC | TNBC | T1 N1 M0 | IIA | 3 | yes | yes | 1,2,3 |
| P61 | IDC | TNBC | T1 N0 M0 | IA | 3 | yes | yes | 1,2,3 |
| P62 | IDC | TNBC | T2 N1 M0 | IIB | 3 | yes | no | 1,2,3 |
| P63 | IDC | TNBC | T1 N2 M0 | IIIA | 3 | yes | no | 1,2,3 |
| P64 | IDC | TNBC | T2 N0 M0 | IIA | 3 | no | yes | 1,2,3 |
| P65 | IDC | TNBC | T2 N3 M0 | IIIC | 3 | yes | yes | 1,2,3 |
| P66 | IDC | TNBC | T1 N1 M0 | IIA | 3 | yes | yes | 1,2,3 |
| P67 | IDC | TNBC | T2 N0 M0 | IIA | 3 | no | no | 1,2,3 |
| P68 | IDC | TNBC | T2 N0 M0 | IIA | 3 | no | yes | 1,2,3 |
| P69 | IDC | TNBC | T1 N0 M0 | IA | 3 | no | yes | 1,2,3 |
| P70 | IDC | TNBC | T1 N0 M0 | IA | 3 | no | no | 1,2,3 |
| P71 | IDC | TNBC | T1 N0 M0 | IA | 3 | no | yes | 1,2,3 |
| P72 | IDC | TNBC | T1 N1 M0 | IIA | 3 | yes | yes | 1,2,3 |
| P73 | IDC | TNBC | T1 N0 N0 | IA | 3 | no | no | 1,2,3 |
| P74 | IDC | ER+/PR+ | T2N0M0 | IIA | 1 | no | no | 2,3 |
| P75 | IDC | ER+/PR+ | T2N0M0 | IIA | 3 | no | no | 3 |
| P76 | IDC | ER+/PR+ | T2N1M0 | IIB | 2 | yes | yes | 3 |
| P77 | IDC | ER+/PR+ | T2N1 | IIB | 3 | yes | yes | 2,3 |
| P78 | IDC | TNBC | T2N0M0 | IIA | 3 | yes | yes | 2 |
|  |  |  |  |  |  |  |  |  |

**Abbreviations and Definitions**

IDC = Invasive ductal carcinoma

ILD = Invasive lobular carcinoma

DCIS = Ductal carcinoma in situ

T1: tumor of 2cm or less

T2: tumor between 2cm and 5cm

T3: tumor more than 5cm

T4: tumor of any size with extension to chest wall or skin

N0: no lymph node involvement

N1: 1-3 lymph nodes involved

N2: 4-9 lymph nodes involved

N3: 10 or more lymph nodes involved

M0 = distal metastasis not present

M1 = distal metastasis present

**Grade:** Modified Bloom Richardson grading scheme based on three morphological features; degree of tumor tubular formation, tumor mitotic activity, nuclear pleomorphism of tumor cells. Higher grade is associated with worse prognosis.

ER: Estrogen receptor

PR: Progesterone receptor

HER-2/neu: Human epidermal growth factor receptor 2

ND: Not determined

Study 1: ALDH1A3 and tPA stained

Study 2: ALDH1A3 and uPA stained

Study 3: ALDH1A3 and PAI-2 stained

**Supplemental Table S4. Families of Proteases or Regulators of Protease Genes Implicated in the Remodeling of the Extracellular Matix**

| **ADAMTS family** | **cathepsins** | **matrix metallo-peptidases** | **membrane metallo-endopeptidases** | **plasminogen activation pathway** | **Trans-membrane serine proteases** | **Metallo-peptidases inhibitors** |
| --- | --- | --- | --- | --- | --- | --- |
| ADAMTS1 | CTSA | MMP1 | MME | PLG | TMPRSS11A | TIMP1 |
| ADAMTS10 | CTSB | MMP10 | MMEL1 | PLAT | TMPRSS11B | TIMP2 |
| ADAMTS12 | CTSC | MMP11 |  | PLAU | TMPRSS11D | TIMP3 |
| ADAMTS13 | CTSD | MMP12 |  | PLAUR | TMPRSS11E | TIMP4 |
| ADAMTS14 | CTSE | MMP13 |  | PLGRKT | TMPRSS11F |  |
| ADAMTS15 | CTSF | MMP14 |  | SERPINB2; SERPINB10 | TMPRSS12 |  |
| ADAMTS16 | CTSG | MMP15 |  | SERPINE1 | TMPRSS13 |  |
| ADAMTS17 | CTSH | MMP16 |  |  | TMPRSS15 |  |
| ADAMTS18 | CTSK | MMP17 |  |  | TMPRSS2 |  |
| ADAMTS19 | CTSL | MMP19 |  |  | TMPRSS3 |  |
| ADAMTS2 | CTSO | MMP2 |  |  | TMPRSS4 |  |
| ADAMTS20 | CTSS | MMP2 |  |  | TMPRSS5 |  |
| ADAMTS3 | CTSV | MMP20 |  |  | TMPRSS6 |  |
| ADAMTS4 | CTSW | MMP21 |  |  | TMPRSS7 |  |
| ADAMTS5 | CTSZ | MMP23A; MMP23B |  |  | TMPRSS9 |  |
| ADAMTS6 |  | MMP23B; MMP23A |  |  |  |  |
| ADAMTS7 |  | MMP24 |  |  |  |  |
| ADAMTS8 |  | MMP25 |  |  |  |  |
| ADAMTS9 |  | MMP26 |  |  |  |  |
|  |  | MMP27 |  |  |  |  |
|  |  | MMP28 |  |  |  |  |
|  |  | MMP3 |  |  |  |  |
|  |  | MMP7 |  |  |  |  |
|  |  | MMP8 |  |  |  |  |
|  |  | MMP9 |  |  |  |  |

**SUPPLEMENTAL FIGURES**

**
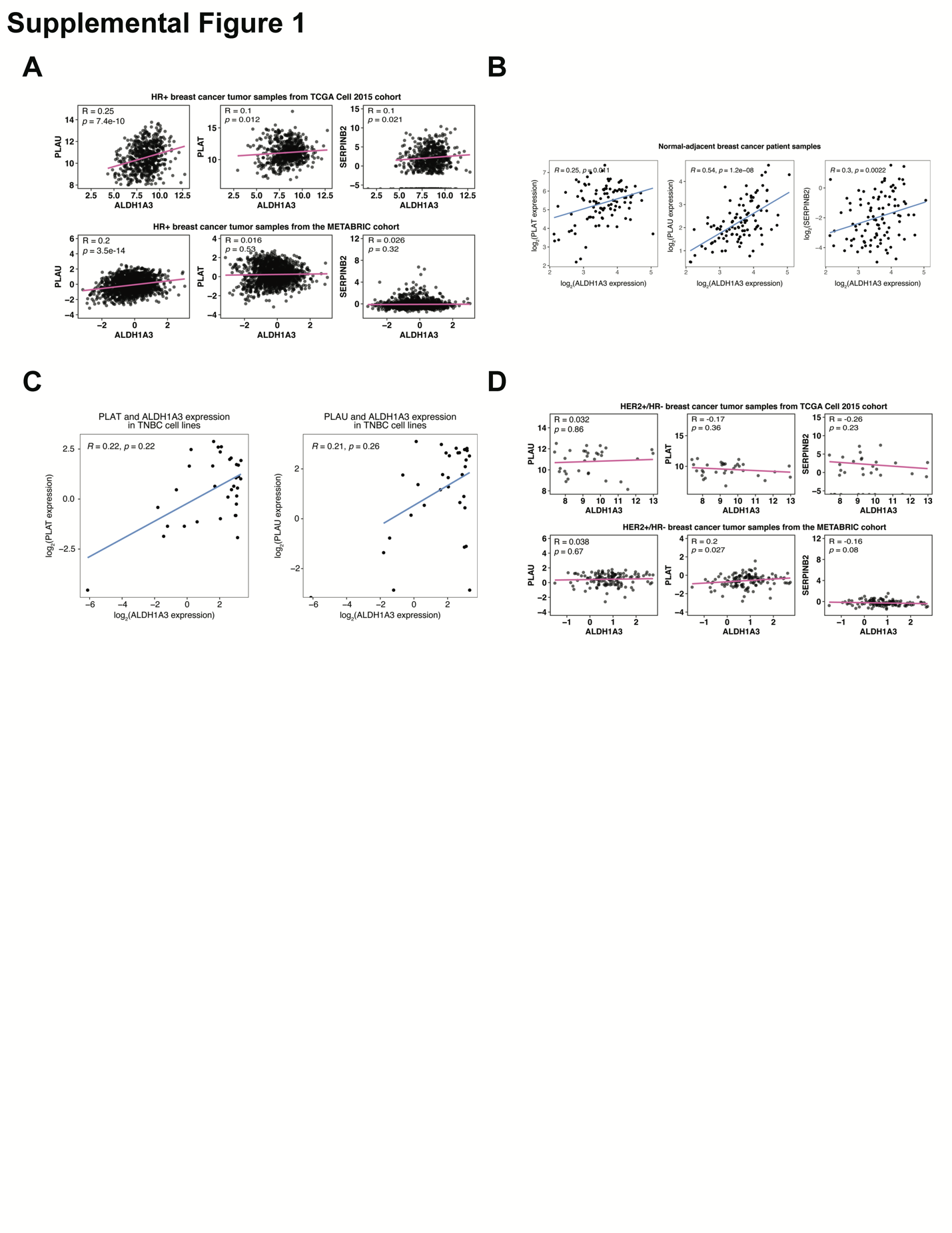
**

**Supplemental Figure S1.** **ALDH1A3 expression correlations with PLAU, PLAT, and SERPINB2 in HR+ breast cancer patient tumors, normal adjacent tissues, TNBC cell lines and HER2+ breast cancer patient tumors. (A)** Expression data for PLAT, PLAU, SERPINB2 and ALDH1A3 from HR+ breast cancer patient tumor samples from the TCGA Cell 2015 cohort (RNA sequencing, top panel) and METABRIC cohort (Z-score, microarray, bottom panel) were obtained from cBioPortal. HR+ patients were identified and extracted (TCGA: n = 593, METABRIC: n = 1478). **(B)** RNA sequencing expression data for PLAT, PLAU, SERPINB2 and ALDH1A3 in normal-adjacent breast cancer patient samples were obtained from the TCGA Firehose cohort accessed through the Broad Institute GDAC portal. Normal-adjacent samples were identified and extracted based on the TCGA tumor barcode (n = 100). **(C)** RNA sequencing expression data for PLAT, PLAU and ALDH1A3 in triple-negative breast cancer (TNBC) cell lines (n = 33) were obtained from the Cancer Cell Line Encyclopedia (CCLE). The Spearman coefficient (R) and corresponding p-value are shown for each correlation. **(D)** Expression data for PLAT, PLAU, SERPINB2 and ALDH1A3 from fHER2+/HR- breast cancer patient tumor samples from the TCGA Cell 2015 cohort (RNA sequencing, top panel) and METABRIC cohort (Z-score, microarray, bottom panel) were obtained from cBioPortal. HER2+ patients were identified and extracted (TCGA: n = 32, METABRIC: n = 127).

**
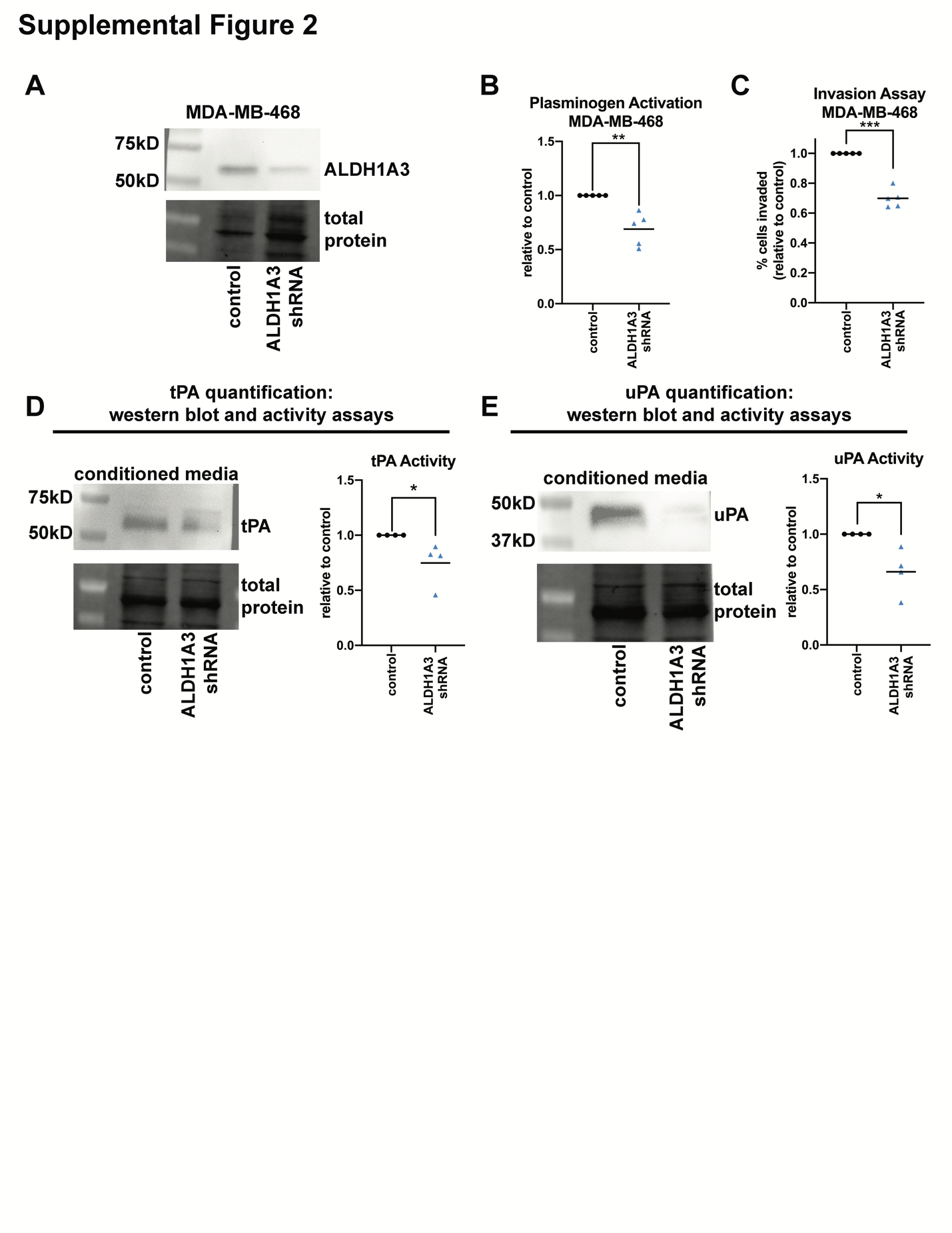
**

**Supplemental Figure S2. Knockdown of ALDH1A3 in MDA-MB-468 cells reduces plasmin, tPA, and uPA activity. (A)** Western blots confirmed reduced expression of ALDH1A3 in MDA-MB-468 cells transduced with ALDH1A3 shRNA2 (Supplemental Table S1). **(B)** The cell surface plasminogen activation assay was performed in MDA-MB-468 cells (shRNA scramble control versus shRNA compared). Values are relative to control cells (n=5). **(C)** Transwell invasion assays were completed with MDA-MB-468 cell (shRNA scramble control versus ALDH1A3 shRNA compared) with FBS as a chemoattractant. **(D, E)** Secreted tPA (**D**) and uPA (**E**) are detected in the conditioned media of MDA-MB-468 cells (shRNA scramble control versus ALDH1A3 shRNA compared) by western blots and activity assays (individual n are shown in the assays). **(B-E)** Significance was determined by one-way ANOVA followed by multiple comparison tests. Significant p values are indicated as follows: * = <0.05, ** = <0.01, *** = <0.001.


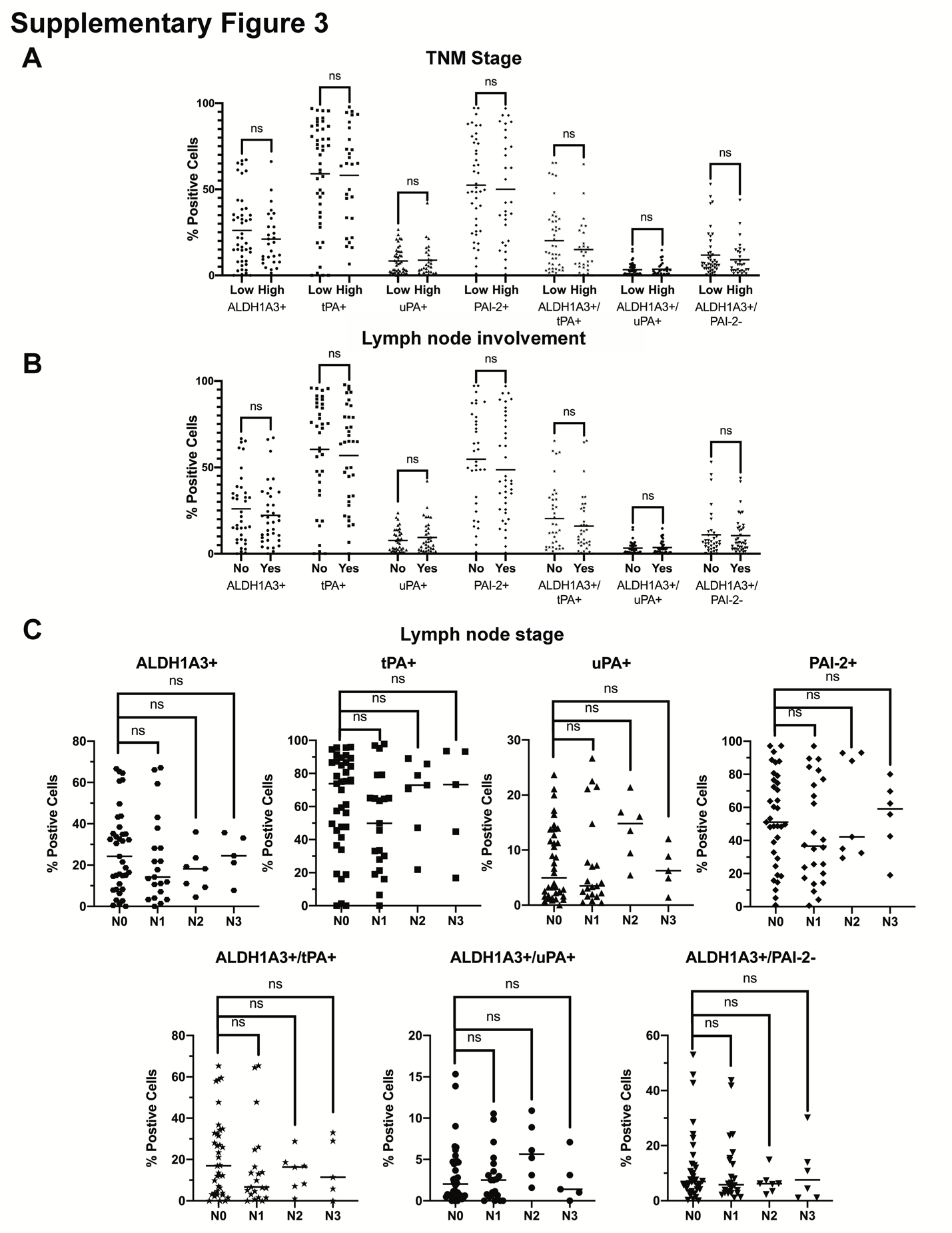


**Supplemental Figure S3. ALDH1A3, tPA, uPA and PAI-2 do not correlate with tumor stage or lymph node involvement.** **(A , B, C)** A panel of formalin-fixed paraffin-embedded 78 breast cancer patient tumor samples (described in **Supplemental Table S3**) are stained for ALDH1A3, tPA, uPA, and PAI-2 for 73 samples and divided into two groups based on **(A)** TNM stage (Low = I, II, IIA stage; High = IIB, IIIA, IIIB, IIIC, IV stage) and **(B)** the presence of lymph node metastasis ( no = N0 lymph node stage, yes = N1, N2 or N3 lymph node stage). **(C)** The patients are divided based on lymph node stage: N0, N1, N2 and N3 and the line represents the median. (**A, B, and C**). Significance was determined by one-way ANOVA followed by multiple comparison tests for experiments, ns = not significant.


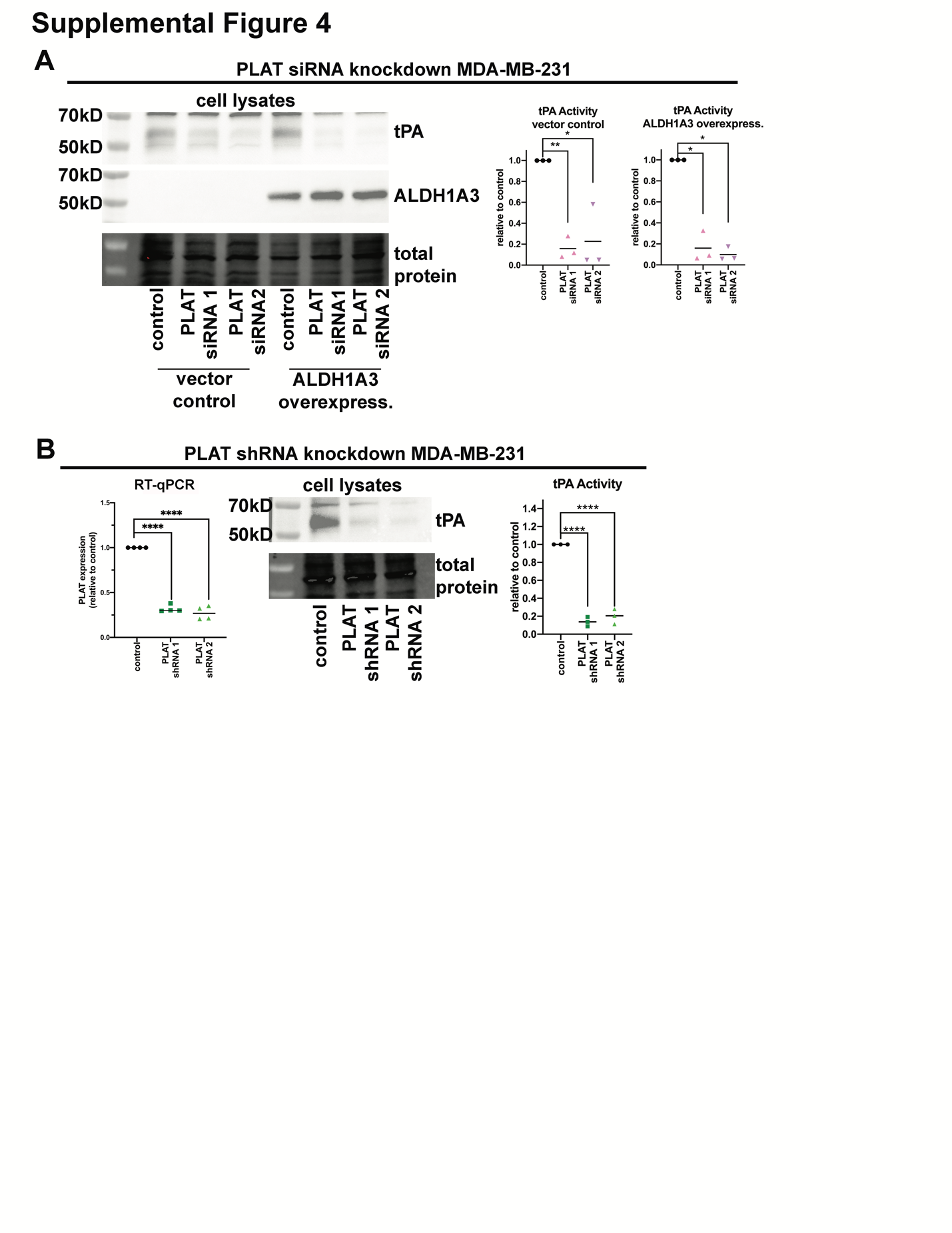


**Supplemental Figure S4.** **PLAT knockdown in MDA-MB-231 cells is confirmed by western blotting and tPA activity assays. A)** Western blot and tPA activity assays confirm knockdown of PLAT by siRNA in vector control and ALDH1A3 overexpressing cells MDA-MB-231 cells). **B)** Western blot and tPA activity assays confirm knockdown of PLAT by shRNA in MDA-MB-231 cells. (**A and B**) Significance was determined through one-way ANOVA followed by multiple comparisons analysis (n=3,4). Significant p values are indicated as follows: * = <0.05, ** = <0.001, **** = <0.0001.

**A Lymph node staining**

**
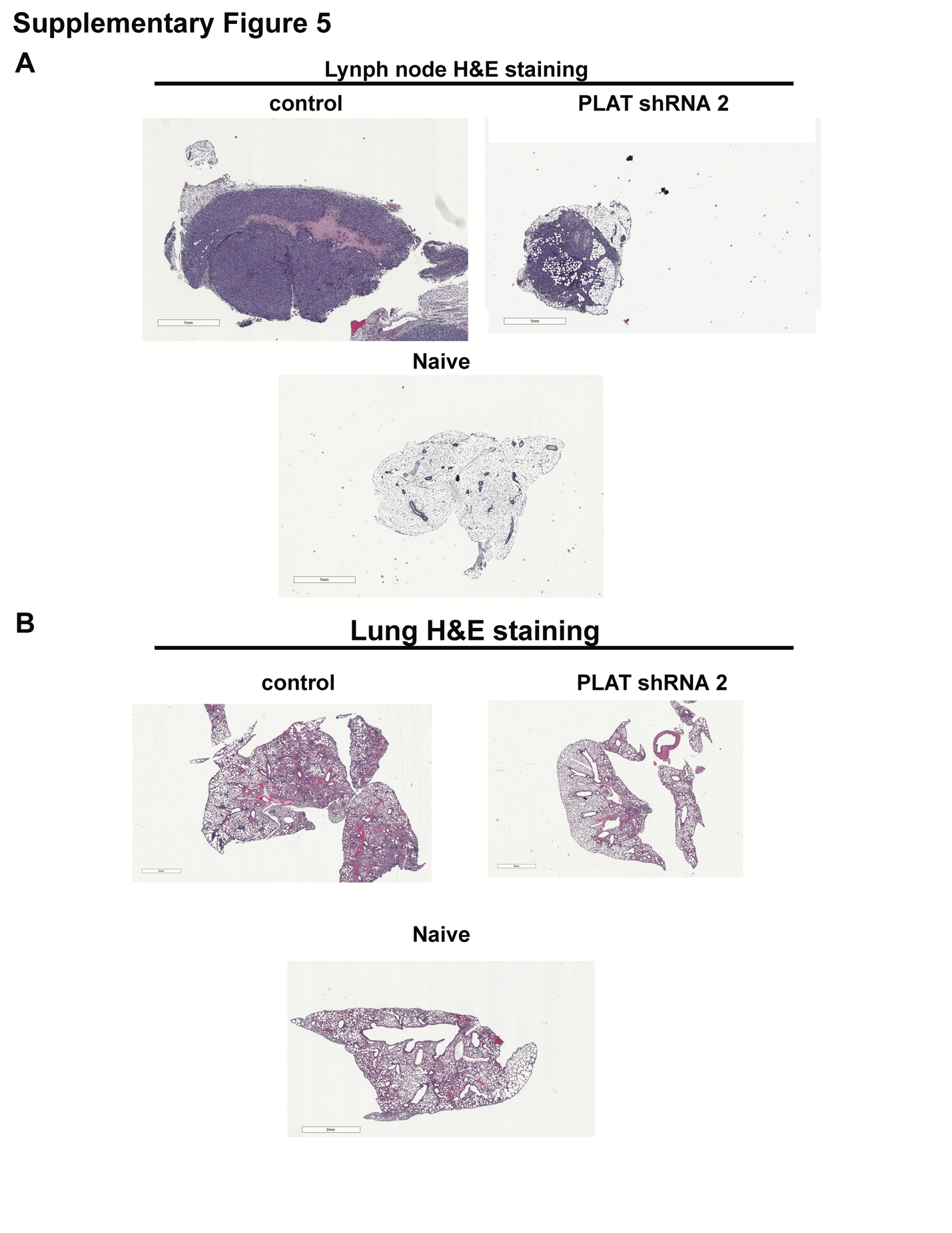
**

**Supplemental Figure S5. The full scanned images of the cropped images from Fig. 7.** The H&E-stained thin sections of the formalin fixed paraffin embedded lymph nodes **(A)** and lungs **(B)** from mice implanted with MDA-MB-231 cells (control and PLAT knockdown) or negative control naïve mouse not implanted with cells. **(A and B)** The scanned images were taken at 20 x magnification and the lymph node images where further magnified 2x. The scale bars are 2mm in the images in **(A)** and 1mm in the images in **(B)**.
